# XXIX^e^ Actualités du Pharo. Sport et santé en milieu tropical - Répercussions des crises (sanitaires, climatiques, sociales, sécuritaires) sur la santé des populations tropicales. 2-4 octobre 2024, Marseille, France: Organisateur : GISPE (Groupe d'intervention en santé publique et épidémiologie) 82 boulevard Tellène, 13008 Marseille, France

**DOI:** 10.48327/mtsi.v5i2.2025.669

**Published:** 2025-04-03

**Authors:** Jean-Paul BOUTIN

**Affiliations:** GISPE, 82 boulevard Tellène, 13008 Marseille, France, www.gispe.org

## Éditorial

Bienvenue à Marseille, au cœur battant de nos fraternités !

Le souffle des Jeux Olympiques et Paralympiques s'est inspiré de notre cher Mistral, ce vent du nord qui pour une fois venait de Paris, pour faire de l’été marseillais un grand moment de fraternité, de joie et d’émotions, débuté le 8 mai avec l'arrivée de la flamme olympique venue de Grèce comme l’étaient les fondateurs de la plus vieille ville de France, et qui n'en finit plus de nourrir nos souvenirs.

Plus d'une cinquantaine de nations méditerranéennes ou tropicales ont eu la joie de voir leurs athlètes monter sur les podiums. Pour la seconde fois dans l'histoire des Jeux ceux-ci se sont déroulés simultanément dans les deux hémisphères, hors et sous les tropiques. En 1956, à l'occasion des jeux de Melbourne (Australie), l’équitation avait eu lieu à Stockholm (Suède) pour des raisons de quarantaine (déjà un sujet sanitaire) et, cette année, les Jeux de Paris se sont exportés jusqu’à Tahiti pour les épreuves de surf.

Des scènes de fraternisation entre athlètes de nationalités différentes, parfois en conflit, ont marqué nos esprits qui voulaient oublier que des nations étaient absentes pour des raisons politiques.

Ces résultats montrent que le sport de très haut niveau est de plus en plus accessible aux jeunes du monde entier et ceci d'abord parce qu'il est plus souvent possible d'arriver à l’âge adulte en bonne santé et d'avoir pu accéder à des sports à l’âge où leurs aïeux devaient travailler. Santé, encadrement et équipements sont le trépied sur lequel la jeunesse du Sud pourra prendre toute sa place dans ces fêtes quadriennales si la paix vient couronner l’édifice. Mais si les sportifs et sportives sont pour beaucoup porteurs d'images de santés éclatantes, la médaille à plusieurs revers. Les blessures parfois évitables, les contre-indications parfois méprisées, le surentraînement, l’équilibre psychologique du sportif, la nutrition, les dopages, les maladies infectieuses liées au milieu où évolue l'athlète sont autant de préoccupations médicales et sanitaires qui doivent être au premier plan et feront l'objet de nos échanges avec des médecins du sport et des sportifs impliqués au Sud et au niveau mondial.

La multiplication des crises, qu'elles soient sanitaires, climatiques, sociales, sécuritaires etc. à toujours eu des conséquences sur la santé des populations vivant en zones tropicales. Ce qui peut paraître comme un truisme pour une crise liée à une épidémie, à la fois cause et conséquence, mérite d’être signifié, décrit, appréhendé pour les autres origines des crises qui affectent les sociétés du Sud. Il est paradoxal de lire encore souvent dès qu'une catastrophe géologique (séisme, tsunami, éruption etc.) survient qu'il faut se précipiter pour enrayer les épidémies et beaucoup moins s'inquiéter des conséquences psychologiques, psychiatriques, nutritionnelles, éducatives, délictueuses et finalement migratoires des crises politiques génératrices de conflits armés ou non. C'est sur ces conséquences de tous les types de crises sur la santé, prise dans sa globalité, pour les populations en zone tropicale que nous consacrerons aussi nos débats de ces 29^e^ Actualités du Pharo en espérant que votre séjour à Marseille vous comblera.

**Figure 1:**
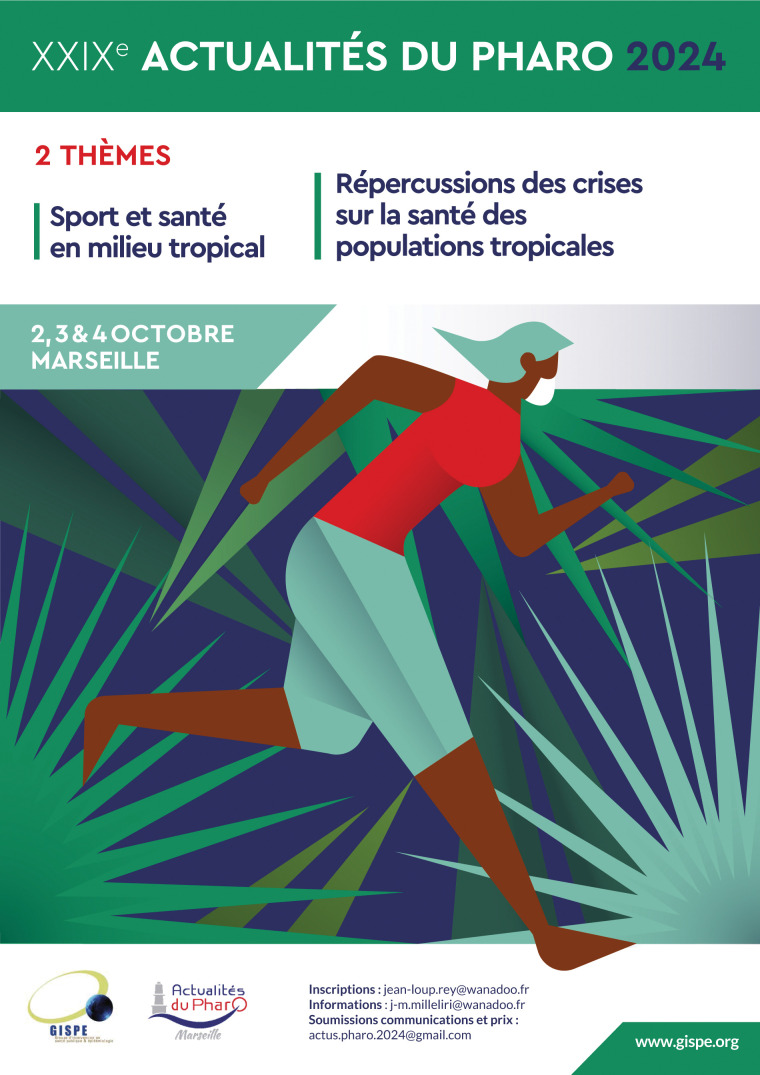
Affiche des XXIX^e^ Actualités du Pharo 2024 Poster of the XXIX^th^ Actualités du Pharo 2024

