# Supplementary material for: XXIXth Actualités du Pharo. Sport and health in tropical environments - Repercussions of crises (health, climate, social, security) on the health of tropical populations. October 2-4, 2024, Marseille, France
Source: Med Trop Sante Int. 2025 Apr 3;5(2):mtsi.v5i2.2025.669. [Article in French] doi: 10.48327/mtsi.v5i2.2025.669 (PMC12308577; doi:10.48327/mtsi.v5i2.2025.669)
Supplement: Related texts

# Research article: L'activité physique pour la santé en Afrique, pourquoi et comment?

**Published:** 2025-04-03

**Authors:** Xavier Bigard

**Affiliations:** Union Cycliste Internationale (UCI), Académie nationale de médecine, médecin du sport, Professeur agrégé du Val de Grâce.

Une activité physique régulière est importante pour la santé et pour le bien-être physique, mental et social de chaque individu, quel que soit son âge. Elle permet d'améliorer la santé mentale et la qualité de vie, de prévenir le développement de facteurs de risques cardio-métaboliques (hypertension, surpoids, obésité) ainsi que la survenue de très nombreuses maladies non transmissibles. Chez l'enfant et l'adolescent, une activité physique régulière est essentielle pour la santé osseuse, la croissance et le développement, et contribue à la réussite scolaire. Au-delà de ces nombreux bienfaits en matière de santé, l'activité physique présente aussi des avantages sociaux, environnementaux et économiques.

En Afrique, on estime qu'un adulte sur quatre (22 % des adultes) et quatre adolescents scolarisés sur cinq (80 % des adolescents) ne sont pas suffisamment actifs. Les niveaux d'inactivité sont régulièrement en hausse à cause entre autres du développement économique et de l'augmentation de la richesse des ménages. Les femmes (26 %) sont moins actives que les hommes (18 %), du fait des normes culturelles et des possibilités plus limitées qui leur sont offertes pour accéder à des lieux de pratique sans danger et financièrement accessibles. L'urbanisation rapide (l'Afrique est l'un des continents qui subit l'urbanisation la plus rapide) et l’évolution des modes de transport couplée au manque d'infrastructures sûres pour la pratique de l'exercice physique ne favorisent pas les déplacements actifs. En Afrique, 44 % des décès dus aux accidents de la route concernent les piétons et les cyclistes.

Quelles solutions ? Il existe de nombreuses façons d’être actif, par la marche, le vélo, le sport, les loisirs actifs, la danse et le jeu. Les pays africains ont adopté la cible définie par l'OMS qui consiste à réduire de 15 % la prévalence de l'inactivité chez les adultes et les adolescents d'ici à 2030.

Les interventions prioritaires à recommander aux différents pays sont les suivantes:

• organiser des campagnes de sensibilisation et diffuser des informations sur les bénéfices de l'activité physique pour la santé, et ce pour toutes les populations;

• renforcer les capacités des agents de santé pour intégrer l’évaluation de l'activité physique ainsi que des conseils de pratique, dans les services de soins primaires;

• investir dans les transports publics, dans les installations sportives publiques et dans des espaces sécurisés qui favorisent la participation à un mode de vie actif;

• renforcer les capacités des enseignants en éducation physique et sportive (EPS) et doter les établissements d'enseignement d'installations de qualité pour la pratique de l'activité physique;

• accroître les investissements dans les clubs sportifs et de loisir qui offrent des possibilités d'activité physique au sein des populations les moins actives (jeunes filles, femmes, personnes âgées).

# Research article: Épreuves sportives d'endurance en milieu chaud et humide : un challenge pour les sportifs, les organisateurs et les professionnels de santé

**Published:** 2025-04-03

**Authors:** Stéphane Bermon

**Affiliations:** World Athletics, médecin du sport

Le changement climatique est une réalité aussi pour le monde du sport. L'apparition plus fréquente de phénomènes météorologiques extrêmes et le réchauffement global posent problème aux athlètes des sports d'endurance quel que soit leur niveau. L'organisation de compétitions comme le marathon ou les compétitions de trial ou *mountain running* représente également un challenge grandissant pour les organisateurs de ces épreuves et leur staff médical. Au premier rang des pathologies sévères possiblement rencontrées dans de telles conditions figurent l'hyperthermie maligne d'effort qui engage le pronostic vital et peut aussi occasionner des séquelles graves à moyen et long terme. La présentation détaillera en particulier les problématiques scientifiques, cliniques, médicales et logistiques autour de l'hyperthermie maligne d'effort du sportif d'endurance.

# Research article: Palestine-RDC, aperçus de la médecine de guerre

**Published:** 2025-04-03

**Authors:** Rony Brauman

**Affiliations:** Fondation Médecins Sans Frontières, médecin et écrivain

République démocratique du Congo (RDC) et Palestine sont deux lieux de crise chronique, deux situations d'intervention médicale humanitaire au long cours, marqués par des épisodes aigus. On s'intéressera aux conditions d'exercice de la médecine dans ces situations, mais aussi et surtout aux conséquences pathologiques très différentes de ces situations. Les deux peuvent être rapprochées par la présence d'un occupant étranger et la présence de groupes armés d'opposition divers, mais se différencient par le contexte politique et social ainsi que par les méthodes de guerre. Ces différences se traduisent en termes épidémiologiques par des profils sanitaires et des besoins d'assistance très contrastés qui seront au centre de la conférence.

# Research article: Impact du réchauffement climatique sur l'expansion des vecteurs et maladies à transmission vectorielle en altitude

**Published:** 2025-04-03

**Authors:** Dominique Jean

**Affiliations:** GISPE, pédiatre infectiologue et himalayiste

Le réchauffement climatique est plus marqué en altitude, ce qui favorise l'expansion des vecteurs et des maladies qu'ils transmettent dans des régions de hauts plateaux ou de montagne précédemment considérées comme indemnes et sans risque. Cela a été observé dans plusieurs pays d'Afrique, en Amérique du Sud et dans les pays himalayens, lors d’études entomologiques et épidémiologiques à haute altitude. La température n'est évidemment pas le seul facteur d'expansion des maladies, mais elle intervient directement sur la survie et la reproduction des vecteurs ainsi que sur le cycle des agents pathogènes parasitaires. L'impact du changement des précipitations est également important mais plus variable dans ses conséquences et selon les pays. De multiples facteurs socio-économiques rendent l’évaluation des risques complexe : migrations, densité de population, urbanisation, pratiques agricoles, déforestation, et surtout systèmes de santé, éventuellement perturbés par les crises politiques ou sanitaires. Des exemples seront présentés pour le paludisme en Tanzanie, Éthiopie et Colombie. Les vecteurs des arboviroses étant de plus en plus signalés en altitude, l'exemple du Népal où les épidémies de dengue ont explosé jusqu’à haute altitude depuis 2019 est emblématique, de même que la présence de l'encéphalite japonaise à 3100 m au Tibet, avec le développement récent d’élevages de porcs. Elle a également été signalée dans des districts de montagne au Népal, où par ailleurs deux autres maladies à transmission vectorielle ont émergé en altitude, retardant l’éradication prévue : leishmaniose viscérale et filariose lymphatique. Surveillance entomologique et épidémiologique, renforcement des systèmes de santé, éducation des populations, lutte antivectorielle, vaccinations ou traitements adaptés, sont indispensables face à l'accélération du changement climatique.

# Research article: Les défis de la santé dans les pays du Sud en guerre

**Published:** 2025-04-03

**Authors:** Marc-Antoine Pérouse de Montclos

**Affiliations:** Institut de recherche pour le développement (IRD), politologue

Cette communication traitera de deux principaux défis de la santé dans les pays du Sud en guerre : le suivi de la surmortalité dans les conflits armés, d'une part, et les attaques contre les personnels de santé, d'autre part. Les exemples seront surtout tirés du Sahel et du Nigeria, pays le plus peuplé d'Afrique. On s'intéressera notamment aux standards de recensement des victimes et aux bases de données disponibles à ce sujet : NigeriaWatch https://www.nigeriawatch.org; Every Casualty Counts https://everycasualty.org; WHO's SSA *(Surveillance System for Attacks on Health Care)*; https://extranet.who.int/ssa/Index.aspx; AWSD *(Aid Worker Security Database)* https://www.aidworkersecurity.org

# Research article: Conférences invitées par la Société francophone de médecine tropicale et santé internationale (SFMTSI)

**Published:** 2025-04-03

**Authors:** Denis Malvy

**Affiliations:** Professeur de maladies infectieuses et tropicales, CHU et faculté de médecine de Bordeaux

Les pays du Sud global connaissent la multiplication de crises de toutes sortes : conflits et guerres hyperboliques, catastrophes naturelles, crises sanitaires ayant en commun les déplacements massifs de populations. Ces évènements s'inscrivent eux-mêmes sur fond de menaces environnementales engageant l'habitabilité à l’échelle continentale, tels l'effondrement de la biodiversité et le changement global. Une illustration est celle de la menace exercée sur l’écosystème forestier du bassin du Congo par les incendies hors normes ou la déforestation. En plus de son impact sur la captation du carbone environnemental, elle représente le levier de la phase pré-épidémique d’émergences zoonotiques. En suivant, la dernière épidémie d'Ebola en République démocratique du Congo a mis à l’épreuve les réponses sanitaires dans des conditions de tension extrême. La région orientale du pays est plongée dans un conflit meurtrier, corollaire de dévastation sociétale, d'effondrement du système de santé, de précarité, de déplacements contraints de populations privées de l'accès aux ressources alimentaires. Bien que les équipes de soins et de suivi des personnes exposées disposent d'outils et d'expérience nécessaires à la riposte, les attaques de la part de groupes armés contre le personnel de santé ont pu en entraver les efforts. Le point chaud du choléra a atteint des niveaux records au Yémen en période de conflit au décours des années 2020. Et les maladies négligées, telles que la leishmaniose ou la trypanosomose humaine africaine, des maladies attendues en voie d’éradication comme la poliomyélite paralytique réapparaissent ou voient leur programme de contrôle suspendus dans les zones d'instabilité ou d’épidémie concomitante. Enfin, l'imbrication des crises porte le fardeau négligé et invisibilisé des psycho traumatismes, et cela dans des populations démunies de services ou programme de santé mentale. Ainsi, des groupes sociaux vulnérables, tels que les enfants et les adolescents âprement meurtris par la guerre sont encore plus marginalisés et maltraités lors de désastres consécutifs. En 2024, de nombreux obstacles pour l'appropriation d'un programme de santé mentale, un droit humain fondamental, sont à franchir dans les pays frappés par toutes sortes de menaces et de calamités.

# Research article: Influence du tremblement de terre et du cyclone sur les infections en Haïti

**Published:** 2025-04-03

**Authors:** Stanislas Rebaudet

**Affiliations:** Infectiologue clinicien et épidémiologiste. Hôpital Européen Marseille, UMR1252 SESSTIM (Aix-Marseille Université, Inserm, IRD)

Dans l'imaginaire collectif, la survenue de l’épidémie de choléra en Haïti en 2010 est souvent attribuée au terrible tremblement de terre survenu quelques mois plus tôt. Cette idée a même été défendue par une partie de la communauté scientifique. On sait aujourd'hui qu'il n'en est rien. Par ailleurs, bien que la transmission du choléra soit fortement impactée par les précipitations, l'impact des cyclones sur le choléra en Haïti s'est avéré beaucoup plus contrastée qu'attendue. Au final, l’étude de l'influence des catastrophes géophysiques et climatiques sur le choléra en Haïti renseigne de manière saisissante les liens complexes entre environnement, contexte social, stratégie de lutte, enjeux politiques, recherche scientifique, et dynamique épidémique.

# Research article: Impact des conflits, des catastrophes et des crises sanitaires sur la prise en charge obstétricale des femmes dans les pays à ressources limitées

**Published:** 2025-04-03

**Authors:** Alexandre Dumont

**Affiliations:** Institut de recherche pour le développement (IRD)

Les conflits armés, les catastrophes et crises sanitaires constituent un problème de santé publique qui pose un sérieux défi aux systèmes de santé des pays du Sud, fragiles et en développement. Les femmes font partie des personnes les plus vulnérables pour de multiples raisons, dont certaines déjà présentes avant les crises. Les violences sexuelles et les viols sont monnaie courante, ce qui contribue à l'augmentation du nombre de grossesses non désirées. Les femmes enceintes font face à une détérioration de l'accès aux services de santé, eux-mêmes sous équipés et mal approvisionnés en médicaments et consommables. Les taux de mortalité maternelle les plus élevés en 2017 concernaient les pays le plus touchés par les conflits armés, comme le Tchad, le Sud Soudan et la Somalie. Le dernier rapport de l'OMS sur l’état de santé de la population mondiale en 2021 suggère une augmentation de la mortalité maternelle et périnatale liée à la pandémie de Covid 19. Des données fiables relatives à la santé des femmes et à la reproduction en temps de crises sont nécessaires pour éclairer les politiques publiques. Des interventions efficaces existent pour limiter l'impact des conflits, catastrophes et crises sanitaires sur la santé maternelle et reproductive.

# Research article: Impact de la pandémie de Covid 19 sur les programmes de prise en charge et d'élimination des maladies infectieuses tropicales

**Published:** 2025-04-03

**Authors:** René Migliani

**Affiliations:** Professeur agrégé du Val-de-Grâce, spécialiste de santé publique

Le virus SARS-Cov-2, responsable de la Covid 19, a provoqué depuis 2020 une pandémie. La riposte a reposé sur plusieurs stratégies : mesures de protection; limitation des interactions sociales; identification et prise en charge des cas; recherche et isolement des contacts et communication pour la mobilisation communautaire.

Fin 2020 des vaccins sont mis à disposition des pays. Le bilan cumulé déclaré de la Covid 19, mi-2024, est de 775 millions de cas, dont 7,1 millions de décès, soit 0,9 % de létalité. L'impact de la Covid 19 en termes de mortalité spécifique estimée fin 2021 était de 15,9 millions de décès, soit 3 fois plus que celle déclarée durant la même période (5,3 millions), avec un recul de l'espérance de vie de 1,8 année.

Les restrictions des déplacements et la réaffectation à la riposte pandémique des ressources des autres programmes sanitaires, ont déclenché une crise globale, dégradant la lutte contre les endémies dans les pays tropicaux aux systèmes de santé fragiles. De 2020 à 2022 dans les pays tropicaux avec la plus forte charge tuberculeuse, l'excès de décès déclarés chez les malades tuberculeux était de 470 000 par rapport à 2019. Le nombre estimé de décès sur cette période était plus de 2 fois supérieur, égal à 1,1 millions. La diminution des thérapies antirétrovirales (TRV) administrées en 2020 a eu un impact direct sur la mortalité VIH. L'excès de décès en 2020-2021 en Afrique, Asie et Amérique latine, en fonction de 50 % de perturbations des activités de lutte a été estimé en moyenne à 300 000 décès. En Afrique, la lutte contre le paludisme a été perturbée en 2020 avec 18 millions de traitements antipaludiques de première ligne non administrés. De 2020 à 2022 la pandémie a été à l'origine d'un excès estimé à 15 millions de cas et 71 000 décès. Les activités de vaccinations des enfants ont nettement diminué sur la planète en 2020. Pour les zones tropicales, le nombre des enfants avec « zéro vaccin » a augmenté d'au moins 10 millions de 2020 à 2022. La pandémie a concerné toutes les maladies tropicales négligées (MTN) notamment par la diminution des distributions de chimiothérapies préventives variant de 12 % à 50 % selon les maladies en 2020. Cet impact est persistant en 2022.

En conclusion, il faudra encore du temps pour préciser l'impact exact de cette pandémie et des moyens importants pour surveiller et gérer les recrudescences de plusieurs maladies et pour rattraper les retards accumulés dans les programmes de lutte, en particulier contre les MTN des régions fragiles des tropiques.

# Research article: Impact des crises sanitaires sur les programmes d'élimination de Maladies Tropicales Négligées : exemple de l'onchocercose et du trachome

**Published:** 2025-04-03

**Authors:** Serge Resnikoff

**Affiliations:** Université de New South Wales (Sydney), Organisation pour la prévention de la cécité, Fondation Théa

Parmi les maladies tropicales négligées dites à chimiothérapie préventive l'onchocercose et le trachome se distinguent par plusieurs aspects, notamment par l'ancienneté de la mise en œuvre des programmes de donation des médicaments (1987 pour l'ivermectine et 1998 pour l'azithromycine) qui ont permis la mise en place d'importants programmes de traitement de masse dans de nombreux pays. Même s'ils sont de nature « verticale » et essentiellement mis en œuvre par les communautés concernées, ces programmes sont intimement intégrés dans les systèmes de santé nationaux et donc soumis aux mêmes contraintes que ceux-ci subissent en cas de crises sanitaires, notamment en termes de priorité, de réorientation des ressources ou de restrictions de mouvements. Dès le début de l’épisode épidémique de Covid 19 des modélisations mathématiques ont cherché à mesurer l'impact, en fonction de la durée, de l'interruption des traitements sur l'atteinte des objectifs d’élimination en tant que problème de santé publique. À ce stade, en 2024, il s'avère que l'impact de cet épisode épidémique s'est traduit par une réduction sensible du nombre de personnes traitées non seulement pendant l’épisode mais aussi depuis la fin de celui-ci. Le nombre de personnes traitées reste ainsi inférieur aux niveaux atteints en 2018-2019. Les raisons de ce déclin sont multiples, incluant la sécurité, l'organisation des campagnes et mêmes les difficultés de production d'azithromycine pour ce qui concerne le trachome. L'impact direct sur l'atteinte des objectifs d’élimination reste apparemment relativement limité.

# Research article: Para sport et santé en Afrique subsaharienne

**Published:** 2025-04-03

**Authors:** Jean-Marie Aleokol Mabieme

**Affiliations:** Fédération camerounaise des sports pour déficients intellectuels (FECASDI), Conseil exécutif des jeux des personnes spéciales de l'Afrique francophone (JPSAF)

Le virage positif du mouvement sportif au troisième millénaire a permis aux peuples de l'Afrique subsaharienne de comprendre l'importance de la pratique sportive pour la santé; ce qui s'explique par la multiplicité des espaces de pratique de l'activité sportive, à savoir le sport pour tous, les espaces des jeux qui sont de plus en plus créés, sans oublier la pratique de l’éducation physique dans les écoles, au secondaire et à l'université. Cette avancée nous laisse dubitatifs quant à l'inclusion, qui voudrait qu'on prenne en compte les personnes en situation de handicap, qui n'ont pas facilement accès à ces infrastructures sportives et même parfois mises de côté lors de l'enseignement de l’éducation physique et sportive. Il ne s'agit pas ici d'une omission, mais d'une hantise qui prend naissance dans la tradition africaine, situant la personne en situation de handicap dans les groupes des personnes maléfiques voire sorcière, qui est punie par la divinité pour sa méchanceté. D'où notre préoccupation suivante : Para-sport et santé publique en Afrique subsaharienne, quelle orientation pour une meilleure inclusion sociale ? Cette préoccupation implique la présentation de l’état de pratique sportive par cette couche sociale vulnérable dans les pays africains, les problèmes rencontrés par les déficients sédentaires et des orientations pour une prise de conscience ou pour une amélioration de la santé de ces personnes par la pratique du para sport.

# Research article: Terang'Aby, le sport au service de l'éducation

**Published:** 2025-04-03

**Authors:** Aby Gaye

**Affiliations:** Association Terang'Aby

Un des objectifs principaux de l'association est de lutter contre la dépigmentation de la peau chez les femmes et principalement les jeunes filles de plus en plus attirées par ce fléau sanitaire.

Grâce à des camps de basket éducatif, les jeunes filles reçoivent des cours dispensés par des professionnels locaux ainsi que des entraînements sportifs.

Depuis sa création, l'association a accompagné plus de 200 jeunes filles en Afrique de l'Ouest. L'Académie Terang'Aby, ouvrira ses portes à la rentrée 2025, 200 jeunes seront accompagnées pendant 3 ans pour suivre une formation complète.

# Research article: Le dopage dans le sport en Afrique : enjeux, challenge et perspectives

**Published:** 2025-04-03

**Authors:** Thomas Capdevielle

**Affiliations:** World Athletics - Athletics integrity unit

L'objet de cette présentation est de présenter l’état de la lutte antidopage en Afrique, continent qui abrite de nombreux champions en athlétisme. Fort de plusieurs années d'expérience sur le terrain, en Afrique de l'Est principalement, nous présenterons la réalité du dopage en Afrique avec en toile de fond les enjeux du sport de haut niveau et sa géopolitique, les challenges rencontrés dans la mise en œuvre d'un programme antidopage sur le continent Africain et les perspectives positives que nous laisse entrevoir l'actualité récente.

# Research article: Bilharziose intestinale chez des athlètes pendant la préparation pour la compétition internationale « Jeux des îles 2023 » à Madagascar

**Published:** 2025-04-03

**Authors:** Dina Ny Aina Liantsoa Randriamiarinjatovo, Seheno Razanatsiorimalala, Bakoly Elie Noro Raholimalala, Milijaona Randrianarivelojosia

**Affiliations:** Unité de parasitologie, Institut Pasteur de Madagascar, BP 1274, Ambatofotsikely Avaradoha - Université de Toliara, Antananarivo, Madagascar

**Introduction.** La 11^e^ édition des Jeux des Iles de l'Océan Indien a eu lieu à Madagascar du 24 août au 3 septembre 2023. Tous les athlètes formant l’équipe nationale malagasy venant de différentes ligues du pays ont été regroupés à Antananarivo pendant la phase préparatoire à partir du 17 juillet 2023. A la demande de la commission médicale de la fédération malagasy d'athlétisme, nous avons réalisé le diagnostic du paludisme et d'helminthiases; et la mesure de l'hémoglobinémie chez les athlètes consentants à 15 jours du début de la compétition.

**Matériels et méthodes.** Pour chaque athlète, des échantillons d'urine et de selles ont été collectés pour la recherche de kystes, œufs et parasites par la microscopie selon les méthodes décrites par l'OMS. A partir d'un échantillon de sang collecté au bout du doigt, un test de diagnostic rapide du paludisme a été réalisé *;* et l'hémoglobinémie a été mesurée avec un hémoglobinomètre. Les athlètes infectés par des vers ont été traités selon la recommandation de la politique nationale en vigueur.

**Résultats.** Chez les 65 athlètes (32 femmes et 33 hommes) âgés de 16 à 52 ans, aucun cas de paludisme et aucun cas de bilharziose urinaire n'a été détecté. La moyenne des valeurs d'hémoglobinémie était de 15,6±1,4 g/dl. Cependant, 20 (30,8 %) athlètes étaient infectés par des vers dont 5 (7,7 %) par *Schistosoma mansoni.* Nous avons mis en évidence *Ascaris lumbricoides, Trichuris trichiura* et *Hymenolepis nana* parfois en coinfection*.*

**Discussion.** Près du tiers des athlètes malagasy qui se préparaient pour une compétition internationale étaient parasités. Le plus surprenantétait la détection de la bilharziose intestinale (deux femmes et trois hommes). Ces athlètes atteints de bilharziose venaient des hautes terres centrales (n = 3), du moyen ouest (n = 1) et de la marge sud des hautes terres centrales (n = 1). Ces résultats sont le reflet du risque d'infection par *Schistosoma* sp dans les zones d'origine des athlètes concernés.

**Conclusion.** La bilharziose et les géohelminthiases étant des problèmes de santé publique à Madagascar, il est recommandé aux responsables de clubs de faire faire régulièrement aux athlètes des examens pour la recherche de kystes, œufs et parasites. La place de la chimiothérapie préventive et le rôle que devrait jouer les athlètes dans leurs communautés respectives afin d'améliorer la lutte contre les helminthiases seront discutés dans notre présentation.

# Research article: Profil des espèces fongiques impliquées dans les mycoses cutanées superficielles à Lomé (Togo)

**Published:** 2025-04-03

**Authors:** Efoe Sossou, A Dorkenoo, AK Adjetey-Toglozombio, FA Lack, A Tagba, A Moukaila, A Dagnra

**Affiliations:** Lomé, Togo

**Introduction.** Les mycoses cutanées superficielles (MCS) sont très fréquentes en dermatologie à Lomé (Togo) mais leur confirmation mycologique est très peu réalisée en routine. L'objectif de cette étude était de décrire le profil des espèces fongiques impliquées dans ces affections à Lomé (Togo).

**Matériel et méthode.** Il s’était agi d'une étude transversale descriptive réalisée de février 2020 à mars 2022, qui a porté sur des patients présentant des lésions suspectes de MCS, reçus en consultation dermatologique dans les trois services de dermatologie de Lomé. Les prélèvements ont été réalisés sur site pour les lésions fortement suspectes de mycoses et analysés au laboratoire de mycologie du CHU Sylvanus Olympio pour une confirmation biologique.

**Résultats.** Au cours de la période d’étude, 565 patients ont été enrôlés et prélevés dont 364 (64,4 %) étaient des femmes avec un âge médian de 31 ans et des intervalles interquartiles (IIQ) de 22 à 41 ans. L'examen direct et/ou la culture a été positive chez 84,7 % (479/565) d'entre eux. Les principales espèces fongiques identifiées étaient les levures dans 63 % dont *Malassezia* sp. 23,8 %, suivies des dermatophytes dans 22,8 % avec pour espèce prédominante *Trichophyton mentagrophytes* (10,8 %), et les moisissures dans 13,1 % dont *Aspergillus niger* et A. *fumigatus* représentant chacun 3,1 %. Les pseudo-dermatophytes n'ont été retrouvés que dans 1 % des cas. Des cas d'associations de mycètes ont été également notés dans 3,5 %.

**Conclusion.** Le spectre des mycètes impliqués dans les MCS à Lomé est dominé par les levures suivies des dermatophytes et des moisissures. Cette diversité de mycètes en cause, impose la réalisation d'un prélèvement mycologique pour une identification du mycète devant toute suspicion de MCS, en vue d'adapter au mieux le traitement antifongique.

# Research article: Pronostic maternel après accouchement par césarienne à l'hôpital régional de Labé (Guinée) : une étude rétrospective de 3 ans (2017-2019)

**Published:** 2025-04-03

**Authors:** Aïssata Tounkara

**Affiliations:** Centre d'excellence d'Afrique pour la prévention et le contrôle des maladies transmissibles (CEA-PCMT), Conakry, Guinée

**Introduction.** La césarienne est une pratique chirurgicale efficace pour sauver la vie des mères et des nouveau-nés, quand son indication médicale est justifiée et pratiquée en temps utile pour les femmes.

**Objectif.** L'objectif général de notre étude était d'analyser le pronostic maternel des césarisées à la maternité de l'hôpital régional de Labé (HRL) de 2017-2019 en Guinée.

**Méthodologie.** Il s'agissait d'une étude descriptive impliquant une revue rétrospective des données de routine des femmes qui ont bénéficié de césarienne à la maternité de l'hôpital régional de Labé entre le 1^er^ janvier 2017 et 31 décembre 2019 soit trois ans.

**Résultats.** L’âge moyen des parturientes césarisées à la maternité de l'hôpital régional de Labé était de 25,58 ans [15-52]. La majorité d'entre elles (N = 2 146, 99,4 %) étaient mariées, nombre d'entre elles (N = 1 338, 62 %), résidaient en milieu urbain. La moitié (1 095, 50,74 %) étaient des ménagères; Les principaux diagnostics en lien avec les indications de la césarienne ont été dominés par les pathologies maternelles et les indications liées aux annexes fœtales, par ailleurs chez certaines ont retrouvait une associationde deux ou trois indications de la césarienne. La suppuration était la principale complication de la césarienne (57, 2,6 %) suivie des complications hémorragiques (15, 0,7 %). Au total 13 décès maternels ont été enregistrés soit un taux de mortalité maternelle de 6/1000. Nous avons retrouvé une association au seuil de 5 % entre l’âge maternel et la parité.

**Conclusion et perspectives.** Cette étude a mis en évidence une brèche sur l'application des mesures d'asepsies et d'antisepsies à la fois en salle d'opération et d'hospitalisation des femmes césarisées, elle s'aligne avec la littérature sur les associations possibles entre l'hémorragie post césarienne et l'atonie utérine.

Ainsi, une sensibilisation des femmes sur le suivi de qualité de la grossesse et le respect des mesures de prévention et de contrôle des infections en milieu hospitalier permettra de réduire les complications liées à la césarienne à la maternité de l'hôpital régional de Labé.

# Research article: PASS-ReL : de la Permanence d'Accès aux Soins de Santé (PASS) vers le système de soins primaires libéral : vécu des malades chroniques précaires de l'Ouest Guyanais en 2022

**Published:** 2025-04-03

**Authors:** Eva Tireau, Camille Obert-Marby

**Affiliations:** Faculté de médecine Hyacinthe Bastaraud - Université des Antilles et de la Guyane - Cayenne

**Introduction.** L'accès aux soins des malades chroniques précaires de Saint-Laurent-du-Maroni est limité par l’éloignement géographique des lieux de soins et l'absence de couverture sociale. Créée en 2020 durant la crise Covid 19, la Permanence d'Accès aux Soins de Santé (PASS) du Centre hospitalier de l'Ouest Guyanais (CHOG) atténue temporairement ces difficultés : consultations médicales délocalisées et accompagnement social, puis les malades obtenant une couverture sociale sont adressés aux médecins généralistes libéraux de la ville.

L'objectif de cette étude menée en 2022 est d'explorer le ressenti des malades chroniques précaires de l'Ouest Guyanais dans l'accès au système de soins primaires libéral après suivi proche de leur domicile et ouverture de droits.

**Méthodologie.** Enquête qualitative par entretiens semi dirigés.

Échantillonnage raisonné avec recrutement de malades chroniques majeurs volontaires anté-rieurement suivis par la PASS du CHOG et réorientés en libéral depuis au moins 6 mois. Entretiens téléphoniques dans la langue du patient en présence d'un médiateur culturel polyglotte du service, avec enregistrement audio. Codage avec le logiciel N Vivo^®^ (Lumivero) et analyse phénoménologique.

**Résultats.** Quatorze patients ont été interrogés. Après suivi à la PASS mobile du CHOG, les malades connaissent mieux leur maladie et désirent un suivi régulier : acteurs, ils choisissent des moyens logistiques et administratifs pour gagner en autonomie dans le système de soins primaires qui semble plus accessible.

Le médecin généraliste devient coordinateur du parcours de soins, et non plus simple prescripteur médicamenteux. Ses compétences relationnelles sont primordiales.

Les aidants naturels sont moins indispensables et la PASS reste une ressource en cas de rupture de droit.

**Discussion et conclusion.** La méthodologie par entretiens téléphoniques avec médiateurs culturels a permis une bonne acceptabilité de l’étude et la libération des paroles et sentiments. Leur intervention, étant source de biais de désirabilité sociale et de confusion sur le motif d'appel, limitée par le recadrage des échanges sur le suivi libéral, a révélé de nouveaux éléments sur la gestion en autonomie des difficultés administratives.

Le suivi à la PASS mobile est un moment charnière pour les malades chroniques précaires de l'Ouest Guyanais : leurs rôle et désirs dans la relation de soins sont profondément transformés. Pour limiter les complications consécutives des ruptures de soins des malades chroniques précaires de l'Ouest Guyanais, le développement de transports, d'une assistance administrative de proximité et de réseaux d’éducation thérapeutique semble indispensable. Ce travail doit être complété par l'exploration du ressenti des médecins libéraux accueillant ces nouveaux patients.

# Research article: Prévenir les récidives de malnutrition infantile : les transferts monétaires sont-ils la solution?

**Published:** 2025-04-03

**Authors:** Vincent Falgairou

**Affiliations:** Croix-Rouge française, 98 rue Didot,75014 Paris, France

**Introduction.** La malnutrition aigüe sévère (MAS) affecte considérablement les enfants de moins de 5 ans, particulièrement dans les régions en crise alimentaire comme Zinder au Niger. Devant l'urgence de cette problématique sanitaire mondiale, des interventions innovantes sont cruciales. Notre étude évalue l'impact des transferts monétaires ciblés sur l'amélioration des scores de diversité et de consommation alimentaire ainsi que sur la réduction des récidives de MAS chez les enfants.

**Méthodes.** Nous avons mené une étude prospective de cohorte exposé/non-exposé, comparant deux groupes de ménages à Zinder en 2022. Les quartiers sélectionnés avaient une prévalence de MAS >10 % durant la période de soudure précédente et les ménages inclus avaient déjà un enfant traité pour MAS. Outre les activités de sensibilisation classiques, le groupe intervention a reçu des transferts monétaires pendant la période de soudure.

**Résultats.** L'analyse a révélé une baisse significative du risque de récidive de MAS, passant de 53,2 % à 36,8 % dans le groupe intervention (p <0,008). Concernant la diversité alimentaire, les scores ont également montré une amélioration significative dans le groupe bénéficiant des transferts monétaires.

**Discussion.** Les résultats indiquent un effet positif global des transferts monétaires sur la prévention de la MAS. Une probable relation dose-réponse a été observée, nécessitant confirmation par des études ultérieures. Nous avons également relevé une possible sous-notification des cas de MAS, en l'absence de carnets de santé dans 23,2 % des cas.

**Conclusion.** Les transferts monétaires peuvent jouer un rôle clé dans la lutte contre la malnutrition infantile. L’étude suggère qu'ils contribuent à améliorer la diversité alimentaire et à diminuer la prévalence des récidives de MAS. Ces résultats encouragent la mise en place de politiques de transfert monétaire dans les contextes de crise alimentaire pour améliorer la santé des enfants. Des recherches plus approfondies sont nécessaires pour optimiser ces interventions et mesurer précisément leur impact à long terme.

# Research article: Rôle de l'infectiologue dans la prise en charge des victimes civiles du conflit armé de Gaza sur le PHA Dixmude : retour d'expérience

**Published:** 2025-04-03

**Authors:** Hélène Savini, Hubert Nielly, Edouard Martin, Matthieu Boye, Paul Chiron, Sarah Amar, Salomé Marty, Frédéric Janvier, Magali Billhot

**Affiliations:** HIA Laveran, BP60149, 13384 Marseille cedex 13, France

**Introduction.** Du 27/11/2023 au 27/01/2024, 124 patients de la bande de Gaza, victimes civiles du conflit armé entre Israël et le Hamas, ont été hospitalisés à bord du Dixmude, porte-hélicoptère amphibie de la Marine Nationale comportant une capacité hospitalière.

**Matériels et méthodes.** Les données démographiques, cliniques, paracliniques et thérapeutiques des patients infectés ont été recueillies et analysées. La capacité de réaliser des cultures bactériologiques et des antibiogrammes n’était pas disponible. Les moyens diagnostiques comprenaient 4 panels de PCR : BCID2, CARBA Xpert SSTI, gastro intestinal et pneumonia panel plus. **Résultats.** Sur un total de 124 patients hospitalisés, 14 patients (dont 5 enfants) présentaient une infection profonde documentée dont 8 infections ostéo-articulaires, 6 infections abdominales et 1 infection du massif facial. La prise en charge avait lieu en moyenne plusieurs semaines après la blessure initiale. Un total de 27 prélèvements profonds étaient positifs. Les infections profondes étaient majoritairement liées à : *Staphylococcus aureus* (n = 11), *Acinetobacter spp* (n = 9), *Pseudomonas aeruginosa* (n = 6), entérobactéries (n = 5). Tous les S. *aureus* étaient résistants à la méticilline. Le gène de résistance CTX-M et le gène NDM étaient détectés pour 11 et 7 bactéries. Le traitement a reposé sur la vancomycine pour 6 patients et sur ceftazidime-avibactam pour 5 patients. Le suivi à long terme était compliqué mais l’évolution à moyen terme était favorable dans tous les cas sauf un.

Parallèlement, les infectiologues ont été confrontés à des infections transmissibles par voie féco-orale : (norovirus, choléra, salmonellose), par voie respiratoire (Covid 19, grippe, VRS), par contact (scabiose, pédiculose).

**Discussion.** Les infectiologues déployés pour cette mission ont été confrontés à deux types d'infections.

La prise en charge multidisciplinaire des infections complexes, notamment ostéo-articulaires, a été rendue difficile par le type et le degré derésistances des bactéries en cause, nécessitant des paris antibiotiques basés sur les détections de gènes de résistance. Aucune désescalade thérapeutique n'a pu être réalisée en l'absence de donnée de sensibilité phénotypique. Les traitements par antibiotiques de dernière génération, dont peu de données existent dans la population pédiatrique, ont été possibles grâce à une adaptabilité du ravitaillement sanitaire.

Ces patients et leurs proches, qui provenaient de Rafah, présentaient également des pathologies communautaires transmissibles dont la gestion a nécessité une adaptation au sein du navire (gestion des excréta et des DASRI, ventilation, isolement géographique).

Ces multiples défis justifient le déploiement d'une expertise en infectiologie lors d'intervention humanitaire au cours de conflit armé.

# Research article: Évaluation de la prise en charge du paludisme non compliqué dans un service d'accueil des urgences

**Published:** 2025-04-03

**Authors:** Cécile Ficko, Pierre-Louis Conan, Dinaherisoa Andriamanantena, Mathieu Cabon, Marie Gominet, Meghann Antoine, Luc Labarbe, Clara Ranc, Virginie Lamand, Sébastien Larréché

**Affiliations:** Service de pathologies infectieuses et tropicales, HIA Bégin, 69 avenue de Paris - 94160 Saint-Mandé, France

**Introduction.** Le paludisme d'importation de l'adulte peut être pris en charge en ambulatoire sous certaines conditions.

**Matériel et méthodes.** Étude rétrospective monocentrique menée dans un centre hospitalier. Tous les adultes pris en charge en ambulatoire du 01/01/2021 au 25/10/2022 pour paludisme non compliqué ont été inclus.

La conformité de la prise en charge a été évaluée (traçabilité des vomissements initiaux, de la mesure du QT corrigé, du traitement prescrit et de ses modalités d'administration, de sa remise au patient et du suivi post-thérapeutique) et a fait l'objet d'une analyse descriptive.

**Résultats.** Vingt-et-un sujets ont été pris en charge pour paludisme non compliqué. L’âge médian était de 30,6 ans, le sex-ratio de 0,75.

Les espèces parasitaires étaient *P. falciparum* (N = 12), *P. ovale* (N = 6), *P. malariae* (N = 2), P. spp (N = 1).

La notion de vomissements initiaux était tracée dans 62 % des cas (N = 13). Un ECG était réalisé dans 20 cas, l'intervalle QTc était tracé sur le compte-rendu des urgences (CRU) ou sur l'ECG lui-même dans 15 cas (71,4 %) et normal dans tous les cas. Trois patients présentaient une hypokaliémie modérée. Aucune adaptation du traitement ou du suivi ECG n’était réalisée chez ces patients, tous traités par ACT.

Les traitements prescrits étaient l'arténimol-pipéraquine dans 13 cas, l'artéméther-luméfantrine dans 4 cas, la chloroquine dans 4 cas. La posologie était adaptée au poids corporel dans 18 cas, non adaptée dans 3 cas. La première prise médicamenteuse était faite aux urgences dans tous les cas, et les contraintes alimentaires étaient notées sur la prescription dans les 17 cas où cela était pertinent. La notion de vomissements après la 1^re^ prise n’était tracée que dans 52 % des cas (N = 11). La traçabilité de la remise de l'intégralité du traitement n’était réalisée que dans 57 % des cas (N = 12). Une ordonnance de traitement était remise à l'ensemble des patients, les horaires de prise étant mentionnés dans 62 % des cas (N = 13), et le respect des contraintes alimentaires chez 11 des 17 sujets pour lesquels cela était pertinent (65 %).

Une ordonnance de suivi biologique était remise à 81 % des sujets (N = 17), et un rendezvous (RDV) de consultation avec un infectiologue était tracé dans le CRU dans 67 % des cas (N = 14). Quinze sujets bénéficiaient d'un suivi au moins partiel en consultation (à J3 J7 ou J28), 6 patients étaient perdus de vue. La comparaison de la proportion de perdus de vue entre le groupe « RDV tracé » et « RDV non tracé » par test de Fischer exact ne mettait pas en évidence de différence significative (p = 0,59).

**Conclusion.** Les recommandations concernant la prise en charge du paludisme non compliqué sont respectées dans cette étude en ce qui concerne le choix des molécules utilisées et l'administration de la 1^re^ prise médicamenteuse au SAU. Des axes d'amélioration sont mis en évidence concernant la traçabilité des vomissements, la mesure du QTc, la conduite à tenir en cas d'hypokaliémie, la traçabilité des horaires de prise et des contraintes alimentaires sur les ordonnances de sortie et du RDV de suivi. Ceci montre l'intérêt de plans d'actions spécifiques et de formations dédiées afin d'améliorer la qualité de la prise en charge du paludisme d'importation.

# Research article: État des lieux et profil épidémiologique de la leishmaniose cutanée à Tlemcen (Algérie), 2019-2022

**Published:** 2025-04-03

**Authors:** Nefissa Chabni, Samia Benmeddah, Zakia Meziane, Samira Benbekhti, Zohra Berrehou, Zineb Yousra Taleb

**Affiliations:** Faculté de médecine, Dr. Benzerdjeb Benaouda, 12 rue, Hamri Ahmed, BP 119, Tlemcen, Algérie

**Introduction.** La leishmaniose cutanée est une maladie parasitaire vectorielle transmise par la piqure d'insecte diptère, le phlébotome. Il s'agit d'un problème de santé publique en Algérie et à Tlemcen, région située à l'ouest algérien.

Cette étude a été réalisée pour déterminer la fréquence de la leishmaniose cutanée diagnostiquée au niveau du Service de parasitologie-mycologie médicales du laboratoire central du CHU Tlemcen.

**Matériel et méthodes.** Il s'agit d'une étude descriptive mixte associant une étude prospective durant une période de 7 mois (du 1^er^ septembre 2021 au 31 mars 2022), et d'une étude rétrospective de 2 ans et 8 mois (de janvier 2019 à août 2021).

Le diagnostic s'est basé sur la visualisation des formes amastigotes de la *Leishmania- sp* par le microscope optique sur des frottis colorés réalisés sur des prélèvements des lésions observées.

**Résultats.** Rétrospectivement, parmi les 106 cas suspects, 21 cas de leishmaniose cutanée (20 %) ont été confirmés, dont la majorité résidait dans la wilaya de Tlemcen (les communes de Sebdou et Laricha sont les plus touchées) ont été confirmés. Prospectivement, 5 cas de leishmaniose cutanée parmi 25 cas suspects (20,8 %) ont été recensés; tous résidaient hors la wilaya de Tlemcen.

Une prédominance masculine dans les deux études a été notée (le *sex-ratio* était de 1,25 et 1,20 respectivement). La tranche d’âge la plus touchée dans l’étude rétrospective était de 20 à 30 ans avec un âge moyen de 27,65±19,3 ans.

Toute les tranches d’âges étaient touchées avec une moyenne de 26,50±18,57 ans prospectivement. Les cas de leishmaniose cutanée étaient essentiellement diagnostiqués en automne et en hiver rétrospectivement et au printemps dans l’étude protective. Rétrospectivement, la plupart des patients présentaient une lésion unique (82,4 %) alors qu‘un patient présentait cinq lésions. Le nombre de lésions retrouvées chez les patients dans l’étude prospective variait de deux à trois localisations. L'aspect ulcéro-crouteux des lésions était dominant dans les deux études (93,3 % et 100 % respectivement).

**Conclusion.** Cette étude a permis de confirmer que la wilaya de Tlemcen et une zone endémique pour la leishmaniose cutanée, avec extension des anciens foyers et apparition de nouveaux foyers, ce qui doit inciter le comité de lutte contre les zoonoses à multiplier et développer des mesures prophylactiques et mettre en œuvre un programme de vulgarisation et de contrôle dans cette régions en fonction des aspect épidémiologiques de la maladies.

# Research article: Impact des conflits armés sur l'élimination de la trypanosomiase humaine africaine dans un foyer endémique en république du Congo

**Published:** 2025-04-03

**Authors:** Ossibi Ibara BR, Adoua Doukaga T, Okoueke R, Nkouka E, Tsota P, Lemboumi S, Effengue S, Assiana R, Kitembo L

**Affiliations:** Programme national de lutte contre la THA, Brazzaville, Congo

**Introduction.** La trypanosomiase humaine africaine (THA) demeure encore un réel problème de santé publique au Congo malgré la baisse significative de l'incidence et de la prévalence de cette endémie sommeilleuse. Elle est là où on la cherche bien. Dans certains départements d'accès difficile comme celui du Pool lors des conflits armés, l'atteinte des objectifs d’élimination de la maladie du sommeil demeure préoccupante.

**Objectif.** Évaluer l'impact des conflits armés dans l'accélération de l’élimination de la THA dans le foyer de Mindouli, département du Pool.

**Patients et méthode.** Étude évaluative, descriptive et analytique des cas de THA dans le foyer de Mindouli entre les périodes pré et post conflits armés dans le département du Pool. La période de conflit allant de 1997-2008 et celle dite post conflit de 2018-2023. Les patients de tout âge, résidant à Mindouli durant la période d’étude et consentant à y participer ont été inclus. Les critères d’évaluation ont été, la population totale examinée (PTE), le nombre de villages prospectés, le nombre de nouveau cas (NC), le nombre de cas traités (NCT) et le taux de létalité selon les périodes.

**Résultats.** Entre 2018 et 2023, la prévalence de la THA est passée de 0,4 % (soit 28 malades identifiés sur une population totale examinée (PTE) de 6 350 et répartis comme suit : 2019 n = 13; 2020-2021 n = 7, 2023 n = 8) versus 0,09 % entre 1997 et 2012 (soit 2 malades identifiés sur unepopulation totale examinée de 2125 et répartis comme suit : 1997 n = 0, 2008 n = 0; 2012 n = 2). Il s'agissait globalement des patients d’âge moyen de 31±3,7 (12-78) ans, ressortissant des villages de Kindamba Ngouedi (n = 1), Missafou (n = 1), Kinkanda (n = 2), Kingoyi (n = 1), Kimbedi (n = 2) et Kingouala (n = 1) (Année 2023). Le diagnostic de la THA était suspecté à la sérologie avec des titration allant de 1/8^e^ à 1/32^e^ et confirmée à la parasitologie chez la plupart des cas. Les cas ont été plus identifiés en période post conflits armés qu'en période de conflits. L'administration du traitement était difficile avec le NECT en période conflictuelle versus en période post-conflit avec le Fexinidazole. La létalité était élevée en période de conflit (100 %) et nul en période post-conflit. L'absence de traitement (p<0,001), de non suivi-post-thérapeutique (p = 0,0001) en lien avec les conflits avait un lien statistiquement significatif avec la survenue de décès.

**Conclusion.** La sécurité ou l'absence de conflits armés facilitant l'accès aux populations résidant dans les foyers endémiques de la maladie du sommeil comme celui de Mindouli est un facteur déterminant dans l'atteinte des objectifs d’élimination de la THA au Congo.

# Research article: Aspects socio-démographiques et professionnels de la coinfection VIH-VHB à Bangui, République Centrafricaine (RCA) : étude transversale en milieu hospitalier

**Published:** 2025-04-03

**Authors:** Gaspard Tékpa, Hermione Dahlia Mossoro-Kpinde, Jessy Guylaine Nazilari, Gertrude Komoyo, Saint Calvaire Henri Diémer

**Affiliations:** Direction de la médecine du travail, Ministère du travail de l'emploi de la protection sociale et de la formation professionnelle - Département de santé publique, Faculté des sciences de la santé - Université de Bangui, République Centrafricaine

**Introduction.** La co-infection VIH-VHB est un problème majeur de santé publique qui n'a pas été suffisamment exploré dans le monde du travail centrafricain. Le but de cette étude était d’évaluer la fréquence de la co-infection VIH-VHB chez les personnes vivant avec le VIH (PVVIH) dans le service des maladies infectieuses et tropicales du Centre hospitalier universitaire de l'Amitié Sino-Centrafricaine de Bangui. **Méthodes.** Une étude rétrospective a été réalisée du 1^er^ janvier 2010 au 31 décembre 2021 au service des maladies infectieuses et tropicales du

CHU Amitié Sino-Centrafricaine. Il comprenait les dossiers de toutes les PVVIH, dont les résultats de la sérologie VHB. Un formulaire standardisé a été utilisé pour collecter les données sociodémographiques et professionnelles par revue documentaire. Les données ont été analysées à l'aide du logiciel Epi-Info 7. Les moyennes, les proportions ont été calculées ainsi que le Chi carré qui était significatif si la valeur p était inférieure à 0,05.

**Résultats.** L’étude a inclus 265 patients, 188 femmes (70,1 %) et 77 hommes (29,1 %), soit un sex-ratio de 0,45. L’âge moyen était de 35,8 ans, plus élevé chez les hommes (40 ans) que chez les femmes (35,8 ans) (p < 0,0001). Les tranches d’âge 25 à 34 ans (37,7 %) et 35 à 44 ans (33,6 %) étaient majoritaires (71,3 %). La majorité des PVVIH étaient au chômage (57,1 %), y compris les femmes au foyer (43,0 %). La prévalence du VHB était de 14,3 %, dont 7,2 % parmi les chômeurs, qui représentent la moitié de toutes les co-infections. La recherche d'associations entre la co-infection VIH-VHB et l'ensemble des caractéristiques sociodémographiques (âge, sexe, état civil) et catégories socioprofessionnelles n'a pas montré de différence significative (p ≥ 0,05). **Conclusion.** Les PVVIH étaient majoritairement des jeunes adultes, des femmes, au chômage; aucune profession n’était significativement associée à la co-infection. La grande majorité des personnes co-infectées n’étaient pas couvertes par le système de santé au travail (chômeurs ou secteur informel). Une action urgente est nécessaire pour améliorer l'accès des travailleurs à la médecine du travail en RCA.

# Research article: Point à date concernant le vaccin contre le Chikungunya

**Published:** 2025-04-03

**Authors:** Prescilla Martinon

**Affiliations:** Valneva, Affaires médicales, 12 ter quai Perrache, Lyon, France

VLA1553 est un candidat vaccin vivant atténué pour l'immunisation active contre la maladie causée par le virus du chikungunya (CHIKV). VLA1553 est basé sur un clone de la souche de La Réunion (CHIKV LR2006-OPY1) et est atténué par la suppression d'une partie du génome du virus. Les principaux résultats cliniques du candidat-vaccin VLA1553 sont présentés dans ce résumé.

Suite à un plan d’études pré-clinique, VLA1553 a été l'objet d’étude cliniques. Un essai de phase 1 randomisé et en aveugle, a révélé destitres d'anticorps neutralisants spécifiques du CHIKV élevés et maintenu dans tous les groupes de doses, ce qui a conduit à la sélection de la dose moyenne pour la suite du développement.

Un essai pivot de phase 3 impliquant 4 115 adultes a atteint son objectif principal, le vaccin candidat VLA1553 induisant une séro-réponse (µPRNT50 ≥150) chez 98,9 % des participants 28 jours après une seule vaccination, qui s'est maintenue pendant au moins 1 an. Le taux élevé de séro-réponse a été observé chez les participants âgés de 18 à 64 ans et chez les adultes âgés de ≥65 ans. VLA1553 a été généralement bien toléré avec un profil d'innocuité acceptable dans les deux groupes d’âge.

# Research article: Plaidoyer pour la défense de la supériorité de l'esprit sur la matière en paludologie comme en toutes choses

**Published:** 2025-04-03

**Authors:** Pierre Druihle

**Affiliations:** Vac4all initiative, 26 Rue Lecourbe, 75015 Paris, France

La présentation sera focalisée principalement sur une analyse des vaccins siamois RTSS et R21, et secondairement sur la tentative récente de traitement du paludisme par anticorps monoclonaux, pour illustrer la dérive technologique qui prévaut depuis plus de 40 ans.

# Research article: Cinétique de l'activité de l'enzyme Glucose-6-Phosphate Déshydrogénase au cours des accès palustres à *Plasmodium vivax* en Guyane

**Published:** 2025-04-03

**Authors:** Laureen Dahuron

**Affiliations:** Université des Antilles et de la Guyane, Faculté de médecine Hyacinthe Bastaraud

**Contexte.** *Plasmodium vivax* est responsable de récurrences par la formation d'hypnozoïtes hépatiques. Pour les éviter, un traitement radical par primaquine est réalisé. La primaquine est contre-indiquée en cas de déficit en Glucose-6-phosphate-Déshydrogénase (G6PD). En Guyane, l'activité de la G6PD est dosée à J14 pour éviter de méconnaître des patients déficitaires en G6PD en contexte hémolytique. Le but de cette étude est d’évaluer la cinétique de la G6PD au cours d'un accès palustre et les facteurs associés à ses variations.

**Méthodes.** Nous avons analysé l'activité de la G6PD entre J1 et J28 chez des patients traités par chloroquine ou dérivé de l'artémisinine au CH de Cayenne entre janvier 2018 et décembre 2020. Les patients ont été répartis dans 3 bras selon le nombre de G6PD dosés disponibles au cours de l'accès palustre étudié. Le bras 1 contient les patients avec plusieurs G6PD dosés, le bras 2 ceux avec un seul G6PD dosé lors de l'accès étudié et une antériorité de G6PD connue, le bras 3 si un seul G6PD disponible.

**Résultats.** Sur les 100 patients inclus dans l’étude de cinétique, 56 sont des hommes avec un âge médian de 31 ans (IQR 19 - 46,8). L'activité de la G6PD varie significativement au cours du temps (p = 0,004) et se stabilise à partir du 3^e^ jour. Le sexe et le taux de réticulocytes ressortent commefacteurs statistiquement associés aux variations de la G6PD; 26,5 % des patients ont été perdus de vue avant traitement par primaquine. Sur les 223 patients inclus dans l’étude de prévalence, aucun ne présente un déficit en G6PD sévère et seulement 1,3 % présentent un déficit entre 10 et 30 %.

**Conclusion.** L'activité de la G6PD varie au cours du temps lors d'un accès palustre à *P. vivax.* Le dosage de la G6PD du troisième jour peut devenir la valeur de référence pour délivrer la primaquine plus tôt afin d'optimiser le traitement radical. Ceci permettrait notamment de diminuer les récurrences précoces et le nombre de perdus de vue.

# Research article: Facteurs associés à la faible couverture du traitement préventif intermittent à la sulfadoxine-pyriméthamine pendant la grossesse dans le district sanitaire de Kangaba en 2022

**Published:** 2025-04-03

**Authors:** Célestin Dembélé

**Affiliations:** Université des Ssiences, des techniques et des technologies de Bamako - Faculté de médecine et d'odontostomatologie, Bamako, Mali

Le paludisme pendant la grossesse est un problème mondial de santé publique. L'OMS recommande dans sa politique de prévention un Traitement Préventif Intermittent pendant la grossesse (TPIg) utilisant la Sulfadoxine-Pyriméthamine (SP). Les résultats de plusieurs études sont en faveur d'une faible couverture de la dose recommandée de la SP malgré d’énormes efforts. C'est dans ce cadre que la présente étude a été menée en vue d’étudier les facteurs associés à l'absorption inadéquate du TPIg-SP.

Nous avons conduit une étude transversale analytique de juin à juillet 2022 auprès de 780 femmes ayant accouché dans les 12 derniers mois dans le district sanitaire de Kangaba. Les données ont été analysées avec le logiciel R (version 4.3.1).

La prise d'au moins trois doses du TPIg-SP était de 26,9 %. Le risque d'absorption de la dose inadéquate était plus élevé en cas de méconnaissance du nombre de doses optimale adéquate (OR = 5,74; [3,76-8,86]; p<0,001) et de l'obtention de la SP ailleurs qu’à la CPN (OR = 2,04; [1,06-4,20]; p = 0,041).

L'utilisation du TPIg-SP demeure faible et est impactée par l'occasion d'obtention et l'ignorance du nombre de doses de SP à prendre.

# Research article: La mucormycose rhino-orbito-cérébrale, à propos d'une observation au service d'infectiologie du CHU de Tizi-Ouzou

**Published:** 2025-04-03

**Authors:** Nadia Brahimi, B. Boulkaria, S. Hadjeb, H. Bouchaib

**Affiliations:** Université Mouloud Mammeri Tizi-Ouzou - Faculté de médecine - Centre hospitalo-universitaire de Tizi-Ouzou, 26 Boulevard Abderrahmane Arous Tizi-Ouzou 15000, Algérie

**Introduction.** Les mucormycoses sont des infections rares, et graves dues à des champignons filamenteux. L'atteinte rhino-orbito-cérébrale est la plus fréquente surtout chez les diabétiques. Le pronostic dépend de la précocité du traitement médical et chirurgical donc du diagnostic microbiologique précoce.

**Matériels et méthodes.** Nous rapportons le cas d'un patient âgé de 68 ans, diabétique depuis 8 ans, admis pour cellulite de l'hémiface droite associée à une paralysie faciale périphérique et du nerf oculomoteur dans un climat fébrile et d'altération de l’état général.

**Résultats.** Examen mycologique nasal par rhi-noscopie permet de montrer de nombreuses colonies de *Rhizopus sp.* Les IRM cérébrales montrent une aggravation des lésions sinusiennes et orbitaire avec perte de substance de la paroi médiane du sinus maxillaire droit.

Traitement : ambisome + posaconazole per os pendant un mois puis posaconazole seul, le traitement chirurgical a été réfuté par l'ORL. L’évolution est favorable après 60 jours de traitement puis complication avec état de choc septique.

**Discussion.** La localisation rhino-orbito-cérébrale est la plus fréquente chez le diabétique. Le diagnostic difficile, le diabète déséquilibré, le retard diagnostic et l'absence de débridement chirurgical sont des facteurs de mauvais

pronostic rapportés et retrouvés chez notre patient. La monothérapie antifongique été retenue dans les protocoles, mais notre patient a été mis sous bithérapie, la supériorité de l'association est en cours d’évaluation.

**Conclusion.** Malgré les thérapeutiques actuelles, le pronostic reste effroyable, avec une mortalité de 80 %, d'où la nécessité d'un diagnostic précoce et un traitement médico-chirurgical rapide et adapté, chez un patient immunodéprimé surtout s'il est diabétique.

# Research article: Profil actuel des zoonoses au service des maladies infectieuses de Batna

**Published:** 2025-04-03

**Authors:** Nabila Kalla

**Affiliations:** Faculté de médecine Batna 2 - Université Batna 2, Batna, Algérie

**Introduction.** Les zoonoses demeurent très fréquentes dans notre région, certaines sévissent à l’état endémique créant un véritable problème de santé publique. L'objectif de ce travail est de décrire les aspects actuels des zoonoses au sein d'un service des maladies infectieuses durant l'année 2022.

**Matériels et méthodes.** Il s'agit d'une étude descriptive rétrospective qui a porté sur les dossiers médicaux des patients hospitalisés dans le service des maladies infectieuses (unité d'hospitalisation homme) pour une zoonose. Les dossiers de ces patients ont été colligés sur une période allant du 1^er^ janvier 2022 au 31 décembre 2022. Les données épidémiologiques, cliniques, paracliniques ont été évaluées.

**Résultats.** Au total, 36 cas de zoonoses ont été recensés. La moyenne d’âge des patients était de 60,2 ans. La plupart des malades avaient au moins un facteur d'exposition au risque. Le délai moyen d’évolution était de 52 jours. Vingt-six de nos patients (soit 71,26 %) avaient une origine rurale. La totalité des zoonoses avaient été documentée sur les données microbiologiques. Les principales zoonoses enregistrées étaient : la brucellose (22 cas), les rickettsioses (2 cas), l'hépatite virale A (5 cas), la leishmaniose cutanée (2 cas), l'hydatidose (2 cas), l'aspergillose (2 cas), la toxoplasmose (1 cas).

**Conclusion.** Sous nos climats, certaines zoonoses sont encore fréquentes. Le renforcement des stratégies de prévention et de lutte contre ces pathologies constitue actuellement l'un des objectifs primordiaux de santé publique.

# Research article: Profil des marqueurs hépatiques chez les patients tuberculeux infectés par l'hépatite B au Centre antituberculeux de Brazzaville

**Published:** 2025-04-03

**Authors:** Nanikaly Moyen, Emmanuel Fonfon Ebata-Mboussa, Faust Réne Okamba Ondzia, Darrel Ornelle Ellion Assiana, Franck Hardain Okemba Okombi

**Affiliations:** Faculté des Sciences de la Santé/Université Marien Ngouabi - 1 Avenue des Jeux Africains - BP 69 – Brazzaville, Congo

**Introduction.** Le métabolisme des antituberculeux (ATB) se déroule dans le foie qui peut être la cible de nombreux virus tel que le virus de l'hépatite B (VHB). L'association tuberculose (TB) et VHB constitue un problème de santé publique. Les médicaments contre la TB (isoniazide, rifampicine, pyrazinamide et éthambutol) et le VHB (ténofovir disoproxil, ténofovir alafénamide et l'entecavir) peuvent perturber les fonctions hépatiques, occasionnant des échecs thérapeutiques. L'objectif de l’étude était de déterminer le profil des marqueurs hépatiques chez les patients tuberculeux sous traitement, infectés par le virus de l'hépatite B.

**Matériels et méthodes.** Il s'est agi d'une étude transversale descriptive menée de juin à novembre 2023 au CAT-BZV. La population était des tuberculeux, des deux sexes, sous ATB, consentant à l’étude et au dépistage du VHB. L'enquête biologique a consisté à détecter l'AgHBs, la charge virale et à doser les transaminases.

**Résultats.** Au total, 219 patients tuberculeux traités ont été inclus, dont 11 patients coïnfectés par le VHB (séroprévalence 5 %). Les patients coïnfectés étaient majoritairement (54,4 %) de sexe masculin et âgé de plus de 35 ans. Ils étaient célibataires (72,7 %) et travaillaient dans le secteur informel (54,5 %). La charge virale était détectée chez 45,5 % patients (5/11). Seul le marqueur hépatique ASAT était perturbé chez une patiente.

**Conclusion.** Nous n'avons pas observé de différence au niveau des transaminases entre patients mono-infectés TB et coinfectés TB/VHB.

# Research article: Prévalence de l'infection par le VHC dans la population générale à la ville de Barika (Algérie)

**Published:** 2025-04-03

**Authors:** Nabila Kalla

**Affiliations:** Faculté de médecine Batna 2, Université Batna 2, Batna, Algérie

**Introduction.** L'hépatite virale C pose un réel problème de santé publique à l’échelle mondiale. En Algérie, sa prévalence est modérée, mais il existe une variabilité géographique dans sa distribution, la willaya de Batna est endémique pour la pathologie. Le dépistage précoce de cette pathologie permet de réduire la morbidité et la mortalité liées à la cirrhose et au carcinome hépatocellulaire. Notre objectif est de déterminer la prévalence de l'hépatite C dans la ville de Barika par la réalisation d'un dépistage dans la population générale.

**Matériels et méthodes.** Une étude descriptive transversale de prévalence de l'hépatite virale C dans la ville de Barika de la willaya de Batna par la réalisation d'une campagne de dépistage de l'hépatite C dans la population générale. Ce dépistage a été organisé pendant 10 jours en mars 2015. Les personnes qui ont été concernées par ce dépistage sont les patients et leurs accompagnateurs qui transitent par les consultations de la polyclinique centrale de Barika pour d'autres pathologies que l'hépatite virale C. Le dépistage a été réalisé par un test rapide immunochro-matographique sur bandelette (les tests rapides d'orientation diagnostique (TROD)). Un test ELISA de confirmation a été réalisé sur les prélèvements positifs.

**Résultats.** 413 personnes ont été dépistées (114 hommes et 299 femmes). Le test rapide d'orientation diagnostique était positif chez 27 patients, soit une prévalence de 6,5 %. La plupart des patients séropositifs sont des femmes (77,8 %). Cette prévalence est plus élevée chez les femmes (7,02 %) que chez les hommes (5,26 %). La tranche d’âge 60-69 ans est la plus touchée 40,70 %, la séroprévalence dans cette classe d’âge est de 12,79 %. Toutes les personnes dépistées séropositives au VHC ont eu un prélèvement sanguin à la recherche de l'ARN VHC par PCR, la PCR VHC est revenue indétectable chez deux patients.

**Conclusion.** Notre campagne de dépistage anonyme et gratuit de l'hépatite virale C a objectivé une estimation de prévalence de l'infection par le VHC dans la ville de Barika à 6,5 %, ce qui a situé notre région dans une zone de forte endémicité de l'infection par le VHC.

# Research article: Évaluation de l'activité de dépistage VIH chez la femme enceinte en consultation prénatale à Tizi Ouzou

**Published:** 2025-04-03

**Authors:** Nadia Brahimi

**Affiliations:** Université Mouloud Mammeri Tizi-Ouzou, Faculté de médecine - Centre hospitalo-universitaire de Tizi-Ouzou, 26 Boulevard Abderrahmane Arous Tizi-Ouzou 15000, Algérie

**Introduction.** Le VIH est un défi majeur pour la santé mondiale, en particulier chez les femmes enceintes. La transmission mère-enfant est évitable grâce au dépistage précoce. Les consultations prénatales sont une opportunité clé de dépistage, mais leur impact est sujet à variations. **Matériel et méthodes.** L’étude prospective est menée sur une période de deux ans, examinant les données de 67 centres de consultations prénatales publics et 72 privés, répartis dans la région. Les caractéristiques sociodémographiques, les facteurs de risques, ainsi que la politique de santé préventive mise en place ont été étudiés.

**Résultats.** 10 253 femmes (24,77 % des naissances attendues) sont dépistées, de 19,4 % la première année à 31,91 % la deuxième année, l’âge moyen est de 32 ans, 0,03 % étaient séropositives, la quasi-totalité sont mariées, de niveau secondaire ou universitaire. Le refus de dépistage n'est que de 4,1 % des cas dû à la cherté du test non remboursé.

**Discussion.** Malgré le programme de santé pour la PTME, le taux de dépistage reste faible par rapport aux directives de l'OMS, l'amélioration des chiffres entre les deux années est due à une sensibilisation plus ciblée, l'acceptabilité du test est satisfaisante, les refus sont motivés pour des raisons économiques.

**Conclusion.** Les résultats de notre étude confirme la faible prévalence de l'infection VIH dans la population de femmes enceinte néanmoins le taux de dépistage reste insuffisant devant les objectifs de l'OMS et du programme national de PTME qui ambitionnent l’élimination de cette contamination verticale par un dépistage généralisé. Un travail de formation de proximité ciblé est à promouvoir.

# Research article: Apport du soutien psychosocial dans la prise en charge des PVVIH en échec thérapeutique suivis au CTA de Donka (Conakry, Guinée)

**Published:** 2025-04-03

**Authors:** Aïssatou Diallo Lamarana Bailo, Joseph Balath, Aïssatou Lamarana Bah, Brigitte Vakou Singa, Mamadou Kanté Djoulde, Mamadou Tounkara Thierno, Mohamed Cisse

**Affiliations:** CHU de Donka, Conakry, Guinée

**Introduction.** L'accompagnement psychosocial est un élément central de la prise en charge globale de l'infection à VIH. La non-observance au traitement ARV favorise l’échec du projet thérapeutique. L’échec thérapeutique survient chez 10 à 20 % des personnes recevant des ARV. L'objectif de notre travail était de décrire l'apport du soutien psychosocial dans la prise en charge des PVVIH en échec thérapeutique suivis au Centre de traitement ambulatoire CTA de Donka.

**Matériel et méthodes.** Il s'agissait d'une étude transversale descriptive d'une durée de 2 ans allant du 1^er^ janvier 2021 au 31 décembre 2022. Nous avons inclus tous les patients en échec thérapeutique (charge virale détectable) adressés par un médecin traitant pour accompagnement psychosocial et suivi au CTA de Donka. Le consentement verbal du client a été obtenu.

**Résultats.** Sur une file active de 3 945 patients, nous en avons colligé 105 en échec thérapeutique soit une prévalence de 2,6 %. Nous avons noté une prédominance féminine soit 61,9 % avec un sex-ratio de 0,6. Ils étaient majoritairement mariés (64,8 %) et résidaient en milieu urbain (78,1 %).

La charge virale moyenne avant l’éducation thérapeutique était de 1 151 200 copies par ml et la charge virale moyenne de contrôle 3 mois après éducation thérapeutique était de 47 188 copies par ml. Les raisons de l’échec thérapeutique étaient dominées par le non-respect de l'heure de prise du médicament chez 43 clients, la prise de médicament traditionnel chez 30 clients, le non-partage du statut sérologique au sein du couple chez 22 clients, l'arrêt volontaire du traitement par survenue d'effets indésirables chez 10 clients.

**Conclusion.** L'accompagnement psycho-social dans le cadre de la prise en charge des PVVIH demeure incontournable pour atteindre les objectifs mais aussi maintenir les patients sous traitements ARV.

# Research article: Mise en place d'une cohorte de couples sérodifférents VIH à l'Hôpital de jour du CHU de Donka, Conakry : retours d'expérience de la Fondation Espoir de Guinée

**Published:** 2025-04-03

**Authors:** Aïssatou Lamarana Bah, Joseph Balath, Brigitte Vakou Singa, Aïssatou Diallo Lamarana Bailo, Souleymane Bah Thierno, Mamadou Kanté Djoulde, Mamadou Tounkara Thierno, Mohamed Cisse

**Affiliations:** Fondation Espoir de Guinée, CHU de Donka, Conakry, Guinée

**Introduction.** En Guinée le dépistage de l'infection par le VIH au sein des couples est fortement encouragé dans les stratégies nationales de prévention. Cependant, l'accompagnement des couples sérodifférents semble présenter certaines limites. Pour améliorer cet accompagnement, l'association de la Fondation Espoir de Guinée (FEG) apporte son appui à l'hôpital de jour du CHU de Donka. L'objectif de cette étude était de partager l'expérience de la FEG dans la mise en place et le suivi d'une cohorte de couples sérodifférents VIH afin de dresser les perspectives pour l'amélioration de cette prise en charge en Guinée.

**Matériels et méthodes.** Il s'agit d'une cohorte prospective suivie entre janvier 2021 et décembre 2022. Durant cette période et lors des séances d'appui psychosocial nous avons proposé à tous les patients vivant en couple de faire venir leurs conjoints/conjointes pour le test de dépistage du VIH en mettant l'accent sur les avantages d'une telle démarche. Pour ceux qui ont accepté de faire venir leurs conjoints, un test rapide VIH a été réalisé sur place après le counseling pré et post-test. À l'issue du test, tous les couples sérodifférents ont été inclus dans notre cohorte.

**Résultats.** Entre janvier 2021 et décembre 2022, nous avons recensé 53 couples sérodifférents VIH sur une file active de 5 379 soit 0,98 %, qui correspondaient à 108 personnes. Parmi elles, il y avait 54 PVVIH dont 35 femmes (64,8 %) et 19 hommes soit 35,2 %. Les PVVIH étaient âgées de 34 ans en moyenne [18 ans et 55 ans]. Elles étaient toutes sous traitement antirétroviral dont 88,8 % sous la combinaison TDF+FTC+EFV et 11,2 % étaient sous TDF+FTC+DTG. La moyenne d’âge des conjoints séronégatifs était de 38 ans [min = 26 ans; max=56 ans], dont 21 femmes soit 38,8 % et 33 hommes soit 61,2 %.

Nous avons noté une bonne acceptation du conjoint positif par le conjoint négatif mais les conjointes négatives acceptent plus facilementleurs conjoints positifs. Parmi les 53 couples, on a retrouvé 2 couples polygames. Ces couples soulèvent des préoccupations de plusieurs ordres : la plupart souhaitant avoir des enfants.

**Conclusion.** Ce travail, premier du genre en Guinée a permis de relever quelques préoccupations des couples sérodifférents VIH notamment le besoin d'une procréation à moindre risque Toutefois, des études qualitatives plus approfondies sont nécessaires pour mieux cerner ces préoccupations.

# Research article: Index de la stigmatisation et la discrimination envers les personnes vivant avec le VIH en Côte d'Ivoire 2.0 (Index Stigma 2.0)

**Published:** 2025-04-03

**Authors:** Anphoumou Geneviève Bah Bile, Eloi Ehui, Léonce Azhiat, Marial Diarrassouba, Nicolas Vako

**Affiliations:** Programme national de lutte contre le sida - ONG Alliance-CI - ONG RIP+ - Abidjan, Côte d'ivoire

**Introduction.** La stigmatisation et la discrimination liées au VIH qui constituent des obstacles majeurs à l'atteinte de l’élimination du sida d'ici 2030, ont été mesurées en 2020 pour les Personnes Vivant avec le VIH (PVVIH) issues de la population générale ainsi que les populations considérées comme celles qui entretiennent la dynamique du VIH.

**Matériels et méthodes.** Cette enquête de 2020 conduite dans les 33 régions sanitaires du Pays a été transversale à visée descriptive intégrant un volet quantitatif et qualitatif avec des focus group et interviews individuelles approfondies. Du 15 octobre au 16 novembre 2020, 2244 PVVIH dont 420 hommes et 1208 femmes, ont été interviewés. Les données ont été analysées sur SPSS® V23.0.

**Résultats et discussion.** Les enquêtés ont été interrogés sur leur ressenti des sentiments d'auto-stigmatisation à cause du statut sérologique au cours des 12 derniers mois.

De manière générale, cette enquête montre que la stigmatisation et la discrimination entraînent un recours moindre aux services de prévention du VIH : non-participation ou participation moindre aux réunions de conseil et d'information sur le VIH et une participation réduite aux programmes de prévention de la transmission mère-enfant.

En outre, les attitudes stigmatisantes vont de pair avec un déni du risque et un recul des comportements préventifs. Dans de nombreux contextes, la peur de la stigmatisation et les croyances stigmatisantes, qui perpétuent l'idée selon laquelle le VIH ne touche que les autres, détournent les personnes du dépistage, des services de soins et de prise en charge.

Il est à souligner que les régions du Cavally (ouest), Gboklê (Sud) et Bélier (Centre) ont plus relevé de cas de stigmatisation/discriminations liées au VIH (entre 30 % et 57 %) qu'au niveau national. Le Goh (centre-ouest) et le Tonkpi (ouest) sont les principales régions où étaient concentrées les expériences de stigmatisation de la part du personnel soignant au cours des douze mois précédant l'enquête (entre 20 et 30 %).

Relativement aux comportements d'auto-stigmatisation, l'on note que ce sont les régions du Lôh Djiboua (90,7 %) et de la Marahoué (75 %) où les taux d'enquêtés qui ont choisi de ne pas participer à des rencontres sociales parce qu'ayant subi une influence négative du fait de la séropositivité sont les plus élevés.

**Conclusion.** Malgré les progrès réalisés, les personnes vivant avec le VIH (PVVIH) restent confrontées à la stigmatisation et à la discrimination liées aux VIH dans les établissements de santé et au sein de la communauté.

# Research article: La promotion de la santé et le bien-être en milieu professionnel et communautaire

**Published:** 2025-04-03

**Authors:** Solange Amethier, Baba Sangare, Mariette Alida Christine Bakou Niangoran, Alexandre Koffi Kouame, Micheline Bonny

**Affiliations:** Ministère de la femme et de la famille, Abidjan, Côte d'Ivoire

La promotion de la santé et le bien-être en milieu professionnel et communautaire sont de plus en plus au centre des engagements au niveau sanitaire et social visant à donner aux individus davantage de maîtrise de leur propre santé et de moyens pour l'améliorer. Notre étude a permis de déterminer le rapport poids/taille ainsi que le taux de sucre dans le sang, le bilan oculaire, dépistage du cancer du sein et du col de l'utérus chez les individus en milieu professionnel et dans la communauté. Des séances d’écoutes, d'entretiens et de consultations médicales ont permis de poser un diagnostic auprès de 257 personnes. L'analyse des séances d’écoute, d'entretien et de consultation ont permis de recenser 18 personnes dont la glycémie est élevée, 4 individus maigres, 66 autres ensurpoids, 90 obèses, 6 souffrants de cataracte, 3 suspectés de glaucome, 91 myopes, 162 ayant des vices de réfraction, 117 cas de photophobies, 5 présentant une myiodésopsie, 120 personnes touchées de presbytie, 33 cas de conjonctivite allergique, 7 personnes souffrant de ptérygion et 71 individus touchés par l'hypermétropie. Les conseils diététiques, la pratique du sport, la vaccination HPV, l'auto-palpation du sein, le dépistage régulier des cancers du sein et du col de l'utérus sont à promouvoir pour avoir une meilleure santé et un bien-être.

# Research article: Enquête de satisfaction des usagers de la Permanence d'Accès aux Soins de Santé et des Équipes mobiles d'accompagnement et de soins du Centre hospitalier de l'Ouest Guyanais en 2022

**Published:** 2025-04-03

**Authors:** Emmanuel Bekolo, Camille Obert-Marby

**Affiliations:** Faculté de Médecine Hyacinthe Bastaraud - Université des Antilles et de la Guyane - Cayenne

**Introduction.** La Permanence d'Accès aux Soins de Santé (PASS) et les Équipes mobiles d'accompagnement et de Soins (EMAS) du Centre hospitalier de l'Ouest Guyanais (CHOG) pallient aux difficultés d'accès aux soins à Saint-Laurent-du-Maroni et alentours en offrant aux populations précaires un accompagnement administratif et social et des soins respectivement en consultations délocalisées et à domicile.

Ces dispositifs innovants ont été récemment déployés durant la crise Covid 19. Une enquête qualitative montre qu'ils répondent à un besoin des malades chroniques, et la fréquentation exponentielle de la PASS déployée en 2020 oblige ce dispositif à se pérenniser et s'adapter. Les EMAS, résultat d'une expérimentation pendant le creux de Covid de l'hiver 2020-2021 ont officiellement été créées fin 2021. Ces deux services n'ont pas encore été évalués.

Cette étude a pour objectif principal de mesurer la satisfaction de leurs usagers en 2022 et pour objectif secondaire de déterminer les domaines d'insatisfaction et proposer des pistes d'amélioration.

**Méthodologie.** Étude prospective uni centrique quantitative par auto-questionnaire. Recrutement de 10 % de l'effectif annuel de chaque dispositif. Les usagers inclus, majeurs,

volontaires, sans comorbidité psychiatrique ou démence, étaient invités en début de vacation PASS ou EMAS à renseigner un auto-questionnaire papier traduit en sept langues, combinant questions à réponses fermées (échelle de Likert) et questions ouvertes et à le remettre en fin de vacation à l'investigateur.

Les données quantitatives ont fait l'objet d'une analyse statistique descriptive exprimée en pourcentages et les données qualitatives issues des questions à réponses ouvertes d'une analyse inductive de contenu thématique.

**Résultats.** Deux-cents usagers de la PASS et 20 patients des EMAS ont participé. 85 % à la PASS et 95 % aux EMAS se disaient très satisfaits. L'insatisfaction concernait les informations sur le fonctionnement des services, l'interprétariat, l'orientation pour problèmes sociaux autres que couverture sociale, la durée en salle d'attente (PASS) et la mise en relation avec les professionnels de santé libéraux (EMAS).

**Discussion et conclusion.** Première évaluation des équipes sanitaires mobiles du CHOG, sa force réside dans son questionnaire minutieusement élaboré et traduit dans les principales langues locales, permettant une forte participation et une bonne représentativité.

L'effectif réduit évaluant les EMAS offre peu de puissance mais donne un aperçu de la satisfaction de ses usagers et sera la base d'une enquête plus importante.

Ce travail montre la forte nécessité de consultations supplémentaires de médecine générale de PASS, et propose des pistes pour améliorer la communication entre usagers, PASS, EMAS, et leurs partenaires médico-sociaux.

# Research article: PrecASOG : vécu des malades chroniques de l'Ouest Guyanais en rupture thérapeutique en 2021

**Published:** 2025-04-03

**Authors:** Maïté Kieffer, Camille Obert-Marby

**Affiliations:** Faculté de Médecine Hyacinthe Bastaraud - Université des Antilles et de la Guyane - Cayenne

**Introduction.** La Permanence d'Accès aux Soins de Santé (PASS) du Centre hospitalier de l'Ouest Guyanais a été créée en urgence durant la crise Covid 19 avec une équipe mobile médicosociale. Depuis 2020, elle propose consultations médicales délocalisées et accompagnement administratif aux personnes précaires de Saint-Laurentdu-Maroni et alentours.

Ce dispositif innovant appartient aux programmes menés par l'Agence régionale de santé dans une logique « d'aller vers » les populations. Après un an, ce service à la fréquentation exponentielle malgré la stabilisation épidémique remarque de fréquentes ruptures thérapeutiques chez les malades chroniques, sans lien évident avec la situation sanitaire régionale.

Cette enquête a pour objectif principal d’étudier le vécu de la rupture thérapeutique par les malades chroniques précaires de l'Ouest Guyanais en 2021.

**Méthodologie.** Étude qualitative par entretiens semi-dirigés. Échantillonnage raisonné avec recrutement lors des vacations en centre-ville ou sites éloignés, et téléphonique, de malades chroniques majeurs consultant le service ayant interrompu leur traitement plus de trois mois. Entretiens réalisés dans la langue du patient avec un médiateur polyglotte du service, en présentiel, avec enregistrement audio. Codage avec le logiciel N Vivo^®^ (Lumivero) et analyse phénoménologique.

**Résultats.** Seize entretiens montrent que la complexité des démarches d'ouverture de couverture sociale, l’éloignement géographique des lieux de soins et les difficultés financières sont les principaux freins à l'observance thérapeutique. Non-ressenti de la maladie, mauvaise compréhension ou effet indésirable du traitement, méconnaissance du système de soins français, ou faille de l'aidant familial sont aussi rapportés.

Les malades en rupture, demandeurs d'accompagnement et de soins, développent des stratégies alternatives : régimes, médecines traditionnelles pour éviter les complications de leur maladie. Le fleuve Maroni, frontière naturelle avec le Surinam, est parfois source de décisions radicales

quant au lieu de vie définitif de certains malades. **Discussion et conclusion.** Première enquête sur la rupture thérapeutique des malades chroniques précaires de l'Ouest Guyanais, c'est une des rares sur leur vécu avec entretiens réalisés dans leurs langues.

Recourir aux médiateurs plutôt qu’à des interprètes entraînait une perte de donnée dans la synthèse et restitution des paroles, limitée par la réécoute des entretiens et relecture des verbatims. Mais dans l'approche phénoménologique ils apportaient leur expérience au contact des différentes communautés et libéraient une parole que l'investigateur seul n'aurait peut-être pas obtenue.

La volonté de soins des malades chroniques précaires de l'Ouest Guyanais doit être encouragée par des actions éducatives (rôle du médecin traitant, démarches administratives) et matérielles (réseau de transport, aide alimentaire, suivi de proximité).

# Research article: Ampleur et déterminants de la surcharge pondérale en milieu universitaire : cas de l'Université Senghor d'Alexandrie

**Published:** 2025-04-03

**Authors:** Ibrahima Bah

**Affiliations:** Agence nationale de sécurité sanitaire – Conakry, Guinée

**Introduction.** Selon l'OMS, la surcharge pondérale se définit comme une « accumulation anormale ou excessive de graisse corporelle pouvant nuire à la santé ». L'obésité connaît actuellement une explosion épidémique touchant non seulement les pays développés mais aussi les pays en développement où elle constitue un sérieux problème de santé publique.

**Objectif.** L'objectif général de notre étude est d’évaluer l'ampleur de la surcharge pondérale chez les étudiants de l'université Senghor d'Alexandrie, ainsi que les facteurs sous-jacents qui lui sont associés.

**Méthodologie.** Une enquête transversale a été menée auprès de l'ensemble des étudiants de la XV^e^ promotion de l'université Senghor. L’évaluation du statut nutritionnel a été réalisée par des mesures anthropométriques. L'analyse statistique des données a permis de déterminer la prévalence de la surcharge pondérale ainsi que les associations entre l’état nutritionnel et les différents indicateurs du mode vie.

**Résultats.** Le surpoids touchait plus du tiers de l'ensemble des étudiants avec une prévalence plus importante chez les étudiants de sexe masculin. L'obésité touche presque un étudiant sur vingt et de façon similaire dans les deux sexes. L'obésité abdominale quant à elle, touchait le tiers des étudiants de sexe féminin et peu répandue chez les étudiants de sexe masculin.

À propos des facteurs de risque, nos résultats ont révélé que le temps d'exposition aux activités sédentaires était plus important que celui consacré aux activités physiques d'intensité élevée; les étudiants de sexe féminin étaient plus sédentaires. L'alimentation des étudiants était beaucoup basée sur les produits d'origine animale, riche en sucres et en matière grasse et pauvre en fruits et légumes.

**Conclusion et perspectives.** La surcharge pondérale à l'université Senghor d'Alexandrie estune réalité. Les habitudes alimentaires et le style de vie influencent la prise de poids. La mise en place d'une étude approfondie pour cibler l’émergence des maladies chroniques non transmissibles associées à l'obésité serait judicieuse.

# Research article: Mortalité des patients atteints de cancer de l'hypopharynx au centre national d'oncologie de Nouakchott (Mauritanie)

**Published:** 2025-04-03

**Authors:** Nah Tolba

**Affiliations:** 99336 Ministère de la Santé, Nouakchott, Mauritanie

**Introduction.** En Mauritanie, le cancer représente 5 % des causes de décès, il a été estimé que 2000 nouveaux cas de cancers sont diagnostiqués chaque année dans le pays et 1500 personnes décèdent chaque année du cancer. Le cancer de l'hypopharynx constitue le deuxième cancer des voies aériennes supérieures. Le diagnostic est souvent tardif. L'objectif de ce travail d’étudier les facteurs associés à la survie des patients ayant un cancer de l'hypopharynx au centre national d'oncologie en Mauritanie.

**Matériels et méthodes.** Une étude transversale et analytique réalisée dans le centre national d'oncologie (CNO) de Nouakchott en Mauritanie entre janvier/2016 à fin décembre/2019 a porté sur 52 patients. Les données ont été recueillies par le biais d'un questionnaire structuré. L'analyse de survie a été effectuée avec un test du Log-Rank. Un modèle de Cox a été utilisé pour estimer le *hazard ratio* et son intervalle de confiance. Des tests bilatéraux ont été réalisés avec un niveau de significativité de 5 %. Les analyses statistiques ont été effectuées à l'aide des logiciels SAS^®^ 9.4 et MedCalc^®^ 20.0.1.0.

**Résultats.** Sur une période de 4 ans, l’étude a porté sur 52 cas de cancer de l'hypopharynx, majoritaires chez les Maures, dans la région de Trarza et chez les 31-40 ans. Le tabagisme et un mauvais état bucco-dentaire étaient des facteurs de risque associés. La dysphagie était le symptôme le plus fréquent et le temps moyen pour consulter était de sept mois. Tous les cas étaient des carcinomes épidermoïdes. La chirurgie avait un but palliatif dans 38,5 % des situations. Plus de la moitié des patients ont reçu de la radiothérapie et de la chimiothérapie. La survie globale à 2 ans était inférieure à 6 %. L'indice OMS (OR = 3,3774 (1,1182 à 10,2016) p = 0,0309), la chimiothérapie (OR = 0,1519 (0,0617 à 0,3741) p<0,0001) et la radiothérapie (OR=0,3378 (0,1619 à 0,7051)) p = 0,0038)) sont statistiquement liés à la survie brute de l'ensemble de la population.

**Conclusion.** Ce travail montre que la survie des patients ayant un cancer de l'hypopharynx est largement liée à l’état général du patient et aux traitements par l'association radiothérapie et chimiothérapie.

# Research article: Thrombose de l'aorte thoracique descendante au cours de la Covid 19 à propos d'un cas

**Published:** 2025-04-03

**Authors:** Zahira Boudiaf, Nassima Djahmi, Amel Touaref, Salima Benyahia Hamidi, Rima Mehamdia, Marwa Krabia, Amine Mammeri

**Affiliations:** Service d'Infectiologie - Service de Microbiologie - CHU d'Annaba, Algérie

**Introduction.** Au-delà d'une maladie infectieuse affectant surtout le tractus respiratoire, il est aujourd'hui clairement établi que la Covid 19 est une maladie thrombogène. En effet 5 % à 30 % des patients hospitalisés manifestent un évènement thrombotique cliniquement avéré.

Selon les données émergentes, une atteinte endothéliale résultant de l'envahissement des cellules par le SARS-Cov-2 et un dérèglement subséquent de la réponse de l'hôte impliquant les voies de l'inflammation et de la coagulation jouent un rôle crucial dans la progression de la Covid 19 sévère.

La thrombose micro et macro-vasculaire contribuerait aux défaillances organiques, à l'atteinte multisystémique et au décès.

Nous rapportons à ce propos une observation d'une pneumonie grave de Covid 19 compliquée d'une thrombose de l'aorte thoracique descendante.

**Observation.** Monsieur MM, âgé de 61 ans, demeurant à Annaba, marié, commerçant de profession, sans antécédents pathologiques particuliers est admis dans notre service le 10/10/2020 pour la prise en charge d'une infection par Sars-Cov2 avec atteinte pulmonaire à la TDM thoracique avec une atteinte > 75 %.

Un diabète inaugural est noté.

La symptomatologie remonte à une semaine avant l'hospitalisation du malade, marquée par l'apparition d'asthénie, courbatures, vertige, nausées, vomissement puis une dyspnée motivant le patient à consulter en ambulatoire où une TDM thoracique a été demandé révélant une pneumonie de type Covid 19 avec une atteinte > 75 %. À l'examen clinique, le patient était conscient, coopérant, apyrétique, mais très asthénique etpolypnéique. Sa pression artérielle à 130/80 mm Hg et sa SaO2 à 87 %. Le reste de l'examen somatique était sans particularité. Le bilan biologique a noté principalement une hyperglycémie (inaugurale) à 21 mmol/l avec un syndrome inflammatoire marqué et un taux de D Dimers à 4342. Le patient a reçu une oxygénothérapie et un traitement médical associant antibiotiques, corticoïde et anticoagulant, selon le protocole national. Une douleur rétro sternale irradiant vers le membre supérieur gauche et une douleur abdominale sont apparues deux jours après son admission.

Une angio-TDM thoracique est alors réalisée mettant en évidence une thrombose artérielle aortique thoracique descendante.

Le patient a été mis sous héparinate sodique mais l’évolution est restée défavorable et le malade est décédé au 9^e^ jour d'hospitalisation.

**Conclusion.** La survenue d'une thrombose artérielle dans un contexte de Covid 19 est un évènement rare qui impose une prise en charge diagnostique et thérapeutique précoce et bien adaptée afin d'améliorer le pronostic sombre qui enveloppe cette complication.

# Research article: Spondylodiscite postrachianesthésie : quelles leçons retenir?

**Published:** 2025-04-03

**Authors:** Khamsa Mokrani, Hamid Hadj Aissa, Rabeh, Aït Hamouda, Soraya Tebbal

**Affiliations:** Service des maladies infectieuses et Faculté de médecine, Batna, Algérie

**Introduction.** Les complications infectieuses secondaires à une rachianesthésie sont rares mais graves. Il s'agit souvent de méningites bactériennes, les spondylodiscites restent exceptionnellement rapportées dans la littérature et sont plutôt décrites dans les suites d'une anesthésie péri médullaire, le plus souvent lorsqu'un cathéter péridural est maintenu plusieurs jours.

Dans le but d'attirer l'attention sur cette complication, nous nous proposons de rapporter un cas observé dans un service de maladies infectieuses.

**Matériels et méthodes.** Patient âgé de 28 ans, admis au service des maladies infectieuses le 06/09/2018 pour syndrome méningé fébrile apparu 48h après résection d'un kyste du cordon spermatique sous rachianesthésie le 26/08/18. La ponction lombaire montre une méningite à *P. aeruginosa.* Le patient est mis sous ciprofloxacine, amikacine et l’évolution est favorable. Après arrêt du traitement à j15 on assiste à une reprise de la fièvre et des céphalées avec apparition de lombalgies. L'IRM cérébromédullaire met en évidence une spondylodiscite L4–L5. Le patient est mis sous ceftazidime et ciprofloxacine. L’évolution est favorable après une durée totale de trois mois de traitement.

**Discussion.** Les spondylodiscites sont exceptionnellement décrites après une rachianesthésie, quelques cas ont été rapportés après chirurgie urologique et orthopédique.

Les praticiens devraient être informés de la possibilité de survenue d'une spondylodiscite dans les jours ou mois suivant une anesthésie spinale et savoir l’évoquer devant toute lombalgie *a fortiori* si fébrile. Les germes en cause sont représentés par les staphylocoques, les BGN et les streptocoques oraux. L'imagerie représente un outil diagnostique majeur. Le pronostic est étroitement lié à une prise en charge précoce.

**Conclusion.** Les spondylodiscites survenant au décours d'une rachianesthésie restent exceptionnelles et les praticiens doivent en être cependant avertis. Leur prévention est primordiale et doit obéir aux règles d'hygiène recommandées par les sociétés savantes. Un diagnostic et une prise en charge précoce permettent d’éviter l’évolution vers des complications pouvant imposer le recours à la chirurgie.

# Research article: Bilharziose intestinale à *Schistosoma mansoni*: à propos d'un cas révélé par une anémie sévère au CHR de Lambaréné (Gabon)

**Published:** 2025-04-03

**Authors:** Madiou Diallo, Dieudonne Eyamame, Davy Tanguy, Mendome Abessolo

**Affiliations:** Service maladies tropicales - Centre hospitalier régional Georges Rawiri, Lambaréné, Gabon

**Introduction.** Parasitose chronique due aux trématodes du genre *Schistosoma* la schistosomiase est un problème majeur de santé publique en Afrique subsaharienne en général et au Gabon en particulier. C'est la deuxième endémie parasitaire mondiale après le paludisme.

Les formes larvaires du ver vivent dans des étangs, des marigots, des rivières et des lacs contaminés par les urines ou des fèces des malades et peuvent pénétrer dans la peau d'une personne lorsqu'elle se baigne ou lors des activités d'agriculture et de pêche.

**Matériels et méthodes.** Nous présentons l'observation d'un patient de 28 ans, diagnostiquéet pris en charge pour bilharziose à *Schistosoma mansoni.* Le patient pécheur, réside dans un village de la Région Centre Sud du Gabon. Il consulte au service des urgences du CHR de Lambaréné, situé à 105 km de son domicile, pour douleur abdominale, fièvre, asthénie, anorexie, nausées, diarrhée glairo-sanguinolente évoluant depuis un mois.

**Résultats.** Aucun antécédent médico chirurgical, cliniquement le patient présentait une profonde altération de l’état général avec asthénie stade II de l'OMS, une pâleur conjonctivale, une sensibilité hypogastrique. Avec de la diarrhée glairo-sanguinolente à l'admission, le patient développe à J2 d'hospitalisation une rectorragie suivie d'anémie sévère. Son taux d'HB chute de 11 g/dl le jour de l'admission à 5,6 g/dl le 5° jour. Hormis les signes indirects du paludisme (PLT à 109 000), le bilan paraclinique a permis le diagnostic de schistosomiase à *Schistosoma mansoni* confirmé par la présence de nombreux œufs de schistosome dans les selles. A l'issue de 8 jours d'hospitalisation et de traitement nous avons noté l'arrêt des symptômes et un taux d'HB de 9,8 g/dl à la sortie.

**Discussion.** La prise en charge par le praziquantel à dose de 40 mg /kg prise unique et la transfusion de deux poches de sang ont jugulé ce cas rare dans notre pratique hospitalière. L'endémicité de la bilharziose dans notre région est à prédominance urinaire due à S. *haematobium.* Les études menées par le Centre de Recherche Médical de Lambaréné ont montré seulement 1 % de schistosomiase intestinale ou rectale due au S. *mansoni.* Le cas de ce patient d’évolution clinique compliquée d'anémie sévère motive dorénavant notre attention sur ces diarrhées avec présence de sang.

**Conclusion.** Le traitement par le praziquantel a été efficace, mais les actions de prévention restent la meilleure arme de lutte, vu le retard de consultation de nos patients.

# Research article: Épidémiologie des gastroentérites bactériennes chez l'enfant dans un hôpital algérien

**Published:** 2025-04-03

**Authors:** Nassima Djahmi, Basma Hamour, Hasna Sehab, Zahira Boudiaf, Amel Bouaricha, Sabrina Amiri, Adnane Otmane, Amina Benali, Sabrina Nedjai

**Affiliations:** BP205 rue Zaafrania 23000 - Annaba, Algérie

**Introduction.** La gastroentérite bactérienne est l'une des principales causes de mortalité et de morbidité de l'enfant dans le monde et elle résulte dans la plupart du temps de l'eau ou d'aliments contaminés. Elles sont courantes dans tous les pays en développement. L'OMS déclare environ 1,7 milliard de cas de diarrhée de l'enfant chaque année dans le monde.

L'objectif de notre travail est d’étudier le profil épidémiologique des gastroentérites bactériennes diagnostiquées au service de Microbiologie du CHU d'Annaba

**Matériel et méthodes.** Il s'agit d'une étude descriptive rétrospective durant une période de neuf ans (2015-2023) portant sur les coprocultures reçues au sein de notre service de patients hospitalisés au CHU d'Annaba. L'identification et l’étude de la sensibilité aux antibiotiques des bactéries isolées était faite par les méthodes conventionnelles et via les automates d'identification et d'antibiogramme. L'interprétation des résultats était faite selon les recommandations du CLSI *(Clinical and Laboratory Standards Institute).*

**Résultats et discussion.** Durant la période suscitée, nous avons colligé 5 047 prélèvements de coproculture. La prévalence des gastroentérites bactériennes diagnostiquées était de l'ordre de 14,5 %. Les bactéries les plus incriminées étaient essentiellement représentées *par Salmonella* avec ces différents sérotypes pathogènes pour l'homme (93 **%**). Suivie *d'Escherichia coli* enté-ro-pathogène (ECEP) et de *Shigella sonnei* qui s'avère la bactérie la plus diagnostiquée dans les diarrhées en 2023. La plupart des souches de *Salmonella heidelberg* isolées étaient productrices de BLSE (92 %), résistantes aux aminosides (90 %), et au cotrimoxazole (81 **%**), avec une forte sensibilité à la ciprofloxacine. La diffusion épidémique de ces souches BLSE avait suscité la réalisation de plusieurs enquêtes épidémiologiques afin de rechercher la source de contamination y compris des enquêtes de dépistage des porteurs sains chez le personnel soignant.

**Conclusion.** Cette étude a démontré le rôle du laboratoire de microbiologie dans la détection et le suivi des épidémies intra-hospitalières à

*Salmonella heidelberg,* ainsi que le signalement de l’émergence des gastro-entérites de *Shigella sonnei* constituant un grand problème de santé publique au CHU d'Annaba.

# Research article: Brucellose ostéo-articulaire : spécificités topographiques et cliniques

**Published:** 2025-04-03

**Authors:** Nabila Kalla

**Affiliations:** Faculté de médecine Batna 2 - Université Batna 2, Batna, Algérie

**Introduction.** La brucellose est une pathologie infectieuse endémique dans notre pays, la localisation ostéoarticulaire est la plus fréquente, dominée par les spondylodiscites et les sacroiléites. Le but de cette étude est de décrire les caractéristiques épidémiocliniques, radiologiques et évolutives de la brucellose ostéoarticulaire.

**Patients et méthodes**. Il s'agit d'une étude descriptive, rétrospective, à partir des dossiers des patients présentant une brucellose ostéoarticulaire hospitalisés au service des maladies infectieuses de Batna. Les dossiers ont été colligés sur une période de deux ans. Le diagnostic de la brucellose ostéoarticulaire a été retenu sur les données de l'imagerie et l’étiologie brucel-lienne a été confirmée par une sérologie et/ou des hémocultures positives et/ou un isolement de brucelles.

**Résultats.** Parmi les 123 patients qui ont été hospitalisés pour une brucellose, 31 (25,2 %) avaient présenté une atteinte ostéoarticulaire. Il s'agit 9 femmes et 22 hommes d’âge moyen de 57,71 ± 13,29 ans. 29 % des cas avaient une profession à risque. Le délai du diagnostic était variable de 30 à 365 jours. L'origine rurale était notée dans 25 cas (80,6 %). Le principal signe révélateur était les rachialgies (96,8 %). Les principaux signes cliniques étaient la fièvre (71 %), les arthralgies (83,9 %) et les myalgies (77,4 %).

Une spondylodiscite a été notée dans 28 (90,3 %) cas, une sacro-iliite dans 4 (13 %) cas, l'association de ces deux localisations a été constaté chez deux patients. Aucun cas d'arthrite n'a été observé.

Pour la spondylodiscite : une épidurite, un abcès des parties molles paravertébrales, un abcès de psoas ont été les principales complications et ont été observées chez 53,6 %, 32,1 %, 10,7 % des patients respectivement. La sacro-iléite était unilatérale dans tous les cas. Le diagnostic étiologique a été confirmé par une sérologie de la brucellose dans tous les cas. La séroagglutination de Wright a montré des anticorps au-dessus de 1/320 dans 71,4 %. Cinq patients avaient des hémocultures positives à *Brucella,* avec deux identifications de *B. melitensis.* La corticothérapie a été prescrite chez 6 (19,4 %) patients. Deux malades ont bénéficié d'un drainage chirurgical. L’évolution était favorable dans tous les cas.

**Discussion.** Nos résultats sont comparables à ceux retrouvés dans la littérature. La spondy-lodiscite brucellienne représente 6 % à 58 % des localisations ostéoarticulaires. Son évolution est généralement favorable, néanmoins des complications neurologiques ont été rapportées.

**Conclusion.** L'atteinte ostéoarticulaire est fréquente dans notre étude. Elle était essentiellement à type de spondylodiscite.

# Research article: Actinomycétome associé à une ostéite du pied à propos d'une observation au service des maladies infectieuses CHU Tizi-Ouzou

**Published:** 2025-04-03

**Authors:** Nadia Brahimi, S. Hadjeb, B. Boulkaria, Morsli, A. Benali

**Affiliations:** Université Mouloud Mammeri Tizi-Ouzou - Faculté de médecine - Centre hospitalo-universitaire de Tizi-Ouzou, 26 Boulevard Abderrahmane Arous Tizi-Ouzou 15000, Algérie

**Introduction.** L'actinomycose est une infection suppurative, due à une bactérie du genre *Actinomyces.* La localisation osseuse est rare. Le tableau clinique est peu évocateur et l’étude bactériologique difficile.

**Matériels et méthodes.** Nous rapportons une observation particulière par ses aspects cliniques, diagnostiques et thérapeutiques chez un patient âgé de 43 ans.

**Résultats.** Notre patient AM âgé de 43 ans, sans antécédents, apiculteur de profession, admis pour complément thérapeutique d'un actinomycétome du pied droit avec ostéite chronique évoluant depuis 6 ans. Le diagnostic étiologique est évoqué à la biopsie après 3 ans d’évolution chronique, confirmé par le prélèvement microbiologique qui a isolé un bacille gram positif anaérobie; ADN *Actinomyces, Actinomadura greaca.* Une IRM du pied retrouve des lésions osseuses, un traitement antibiotique a été prescrit par amoxicilline pendant 9 mois, puis itraconazole pendant 3 mois, puis reprise de l'amoxicilline pendant 5 mois devant l'absence d'amélioration clinique. Une sanction chirurgicale a été discutée, mais réfutée par le chirurgien en faveur d'un traitement médical d'une année, le patient est toujours sous surveillance clinique.

**Discussion.** L’évolution traînante chez notre patient rend compte des difficultés d’évoquer précocement le diagnostic et de le confirmer, surtout que l'exposition professionnelle est possible, l'atteinte osseuse est rare selon la littérature, le retard thérapeutique est préjudiciable sur le plan fonctionnel, la sanction chirurgicale sera de dernier recours selon certaines données, des évolutions favorables ont été obtenues par antibiothérapie prolongée.

**Conclusion.** L'actinomycose est une infection chronique rare, qu'il faut savoir évoquer devant toute lésion traînante cutanée et osseuse, des prélèvements microbiologiques et biopsiques doivent être réalisés pour un traitement précoce.

# Research article: Les mycétomes au Sénégal : impact de la dynamique pluviométrique de 1947 à 2022 sur le profil épidémiologique et étiologique à propos de 291 cas

**Published:** 2025-04-03

**Authors:** Saër Diaidié

**Affiliations:** Université Cheikh Anta Diop de Dakar – BP 5005 - Dakar, Sénégal

**Introduction.** Une faible pluviométrie est le principal facteur de risque des mycétomes. Au Sénégal, les foyers de mycétomes jusqu’à ce jour connus sont le nord, le centre et l'ouest dans une moindre mesure. Suite au changement climatique et son impact sur la pluviométrie, nous avons jugé opportun d’évaluer la nouvelle cartographie des mycétomes au Sénégal.

**Méthodologie.** Il s'agissait d'une étude rétrospective, descriptive colligeant les cas de mycétomes (2000-2023) suivis dans les services de référence de Dermatologie et d'Orthopédie des CHU de Dakar et des hôpitaux régionaux. Les données sur les isohyètes (1947-2022) ont été fournies par l'Agence Nationale de l'Aviation Civile et de la Météorologie. Les tests statistiques étaient faits par EpiInfo (seuil de significativité p≤0,05).

**Résultats.** L'analyse de la pluviométrie a montré une baisse de la moyenne nationale de 200 mm. L'isohyète 800, jugé seuil pluviométrique des mycétomes, s'est déplacé de 200 km vers le sud. La Casamance (sud) autrefois arrosée entre 1 100 et 1 600 mn enregistre depuis 1993 une pluviométrie annuelle entre 800 et 1 200 mm. Nous avons colligé 291 cas. Il ressort de notre étude que des mycétomes existent au-delà de 800 mm. Des cas autochtones ont été notés en Casamance (n = 7). Les mycétomes fongiques représentent près de la moitié des cas (49,8 %, n = 155) et sont davantage à grains noirs *(Madurella mycetomatis)* et statistiquement corrélés à une pluviométrie comprise entre 200 et 400 mm (p = 0,01) correspondant à la zone nord. Les actinomycétomes sévissaient en majorité au-delà de 400 mm (p = 0,01).

**Discussion.** Le changement climatique par le biais d'une baisse pluviométrique a élargi l'aire des mycétomes au Sénégal. Notre étude montre une croissance sans cesse des eumycétomes tendant à égaler les formes bactériennes qui jadis représentaient plus de 2/3 des cas. De plus elle révèle que plus la pluviométrie est faible, plus fréquents sont les eumycétomes. L'augmentation de ces derniers au traitement indisponible et onéreux est inquiétante. Le coût du traitement annuel est estimé à 3970 euros, très au-delà des moyens financiers des malades le plus souvent issus de milieux défavorisés. Aucun programme ne vise à ce jour d'alléger ce fardeau vécu par les patients. Il s'agit d'une véritable négligence de malades présentant une Maladie Tropicale Négligée.

**Conclusion.** Le changement climatique a élargi la cartographie des mycétomes au Sénégal. L'accroissement des cas d'eumycétomes nécessite d'orienter la politique de lutte sur la recherche d'alternative thérapeutique et le subventionnement de l'itraconazole. L'application immédiate des décisions issues des Conférences des Parties (COP) est plus qu'impérieuse afin d'améliorer le « *One Health* ».

# Research article: Un aperçu complet du développement du vaccin vivant atténué contre la dengue, TAK-003

**Published:** 2025-04-03

**Authors:** Walid Kandeil, Sharma Sharma

**Affiliations:** Takeda France, 112 Avenue Kléber 75116 Paris

**Introduction.** Le nombre de cas de dengue augmente, ce qui rend nécessaire le développement urgent d'un vaccin sûr et efficace. Cependant, la mise au point d'un vaccin vivant atténué contre la dengue a rencontré plusieurs défis, tels que l'induction de réponses immunitaires tétravalentes multifacettes, l’équilibre entre la sécurité et l'immunogénicité par une atténuation virale optimale, et l'obtention de suffisamment de données au niveau des sérotypes grâce à des analyses à long terme de l'efficacité et de la sécurité dans différents pays. Nous présentons un aperçu du programme de développement du vaccin vivant atténué tétravalent contre la dengue de Takeda (TAK-003) et partageons des approches pour surmonter les principaux défis.

**Matériels et méthodes.** Des investigations précliniques approfondies et 19 essais cliniques (28 175 participants, de 1,5 à 60 ans, provenant de 13 pays) ont soutenu le développement du TAK-003. Les études ont inclus l'analyse de la formulation du vaccin, du calendrier de doses, de la sécurité et de l'immunogénicité, conduisant à l'essai pivot de phase III DEN-301 (NCT02747927).

**Résultats.** Les études initiales ont confirmé la stabilité génétique, une atténuation optimale avec une réactogénicité acceptable, et une immunogénicité équilibrée. L'essai DEN-204 (NCT02302066) a soutenu le calendrier à deux doses pour maximiser la séroconversion multivalente chez les receveurs du TAK-003.

L'essai DEN-301 a montré que l'efficacité du vaccin était de 80,2 % (IC à 95 % : 73,3-85,3) contre la dengue virologiquement confirmée (DVC) après 12 mois (critère d’évaluation principal) et après 18 mois de 90,4 % (IC à 95 % : 82,6-94,7) contre l'hospitalisation. L'efficacité du vaccin contre la DVC et l'hospitalisation était comparable entre les vaccinés séronégatifs et ceux déjà exposés à la dengue. L'efficacité cumulative sur 54 mois était de 61,2 % (IC à 95 % : 56,0-65,8) contre la DVC et de 84,1 % (IC à 95 % : 77,8-88,6) contre l'hospitalisation, bien que variable selon le sérotype. Aucun signal de sécurité cliniquement important, indépendamment du statut sérologique de base/âge, n'a été observé par l'analyse de sécurité intégrée.

**Conclusion.** Le vaccin TAK-003, développé à travers des études approfondies, a montré une réactogénicité acceptable, une immunogénicité équilibrée, une efficacité et une sécurité prolongées, répondant à un besoin non satisfait pour un vaccin contre la dengue sans risques de sécurité cliniquement importants identifiés.
